# Psychosis prevalence and physical, metabolic and cognitive co-morbidity: data from the second Australian national survey of psychosis

**DOI:** 10.1017/S0033291713002973

**Published:** 2014-01-02

**Authors:** V. A. Morgan, J. J. McGrath, A. Jablensky, J. C. Badcock, A. Waterreus, R. Bush, V. Carr, D. Castle, M. Cohen, C. Galletly, C. Harvey, B. Hocking, P. McGorry, A. L. Neil, S. Saw, S. Shah, H. J. Stain, A. Mackinnon

**Affiliations:** 1 Neuropsychiatric Epidemiology Research Unit, School of Psychiatry and Clinical Neurosciences, The University of Western Australia, Crawley, WA, Australia; 2 Centre for Clinical Research in Neuropsychiatry, School of Psychiatry and Clinical Neurosciences, The University of Western Australia, Crawley, WA, Australia; 3 Queensland Brain Institute, The University of Queensland, Brisbane, QLD, Australia; 4 Queensland Centre for Mental Health Research, Brisbane, QLD, Australia; 5 School of Psychology, The University of Western Australia, Crawley, Western Australia; 6 Clinical Research Centre, North Metropolitan Health Service-Mental Health, Mount Claremont, WA, Australia; 7 School of Population Health, The University of Queensland, Ipswich, QLD, Australia; 8 School of Psychiatry, The University of New South Wales, Sydney, NSW, Australia; 9 Schizophrenia Research Institute, Sydney, NSW, Australia; 10 Department of Psychiatry, The University of Melbourne, Melbourne, VIC, Australia; 11 St Vincent's Hospital, Melbourne, VIC, Australia; 12 Hunter New England Mental Health, Newcastle, NSW, Australia; 13 School of Medicine and Public Health, The University of Newcastle, Newcastle, NSW, Australia; 14 School of Medicine, University of Adelaide, Adelaide, SA, Australia; 15 Ramsay Health Care (SA) Mental Health Services, Adelaide, SA, Australia; 16 Northern Sector, Adelaide Metro Mental Health Directorate, Adelaide, SA, Australia; 17 Psychosocial Research Centre, North West Area Mental Health Services, Coburg, VIC, Australia; 18 SANE Australia, Melbourne, VIC, Australia; 19 Orygen Youth Health Research Centre, Melbourne, VIC, Australia; 20 Centre for Youth Mental Health, The University of Melbourne, Melbourne, VIC, Australia; 21 Menzies Research Institute Tasmania, University of Tasmania, Hobart, Australia; 22 Australian Government Department of Health and Ageing, Canberra, ACT, Australia; 23 Centre for Rural and Remote Mental Health, University of Newcastle, Newcastle, NSW, Australia; 24 School of Medicine, Pharmacy and Health, Durham University, Durham, UK

**Keywords:** Bipolar disorder, schizo-affective disorder, schizophrenia, speed of information processing, substance abuse

## Abstract

**Background:**

There are insufficient data from nationwide surveys on the prevalence of specific psychotic disorders and associated co-morbidities.

**Method:**

The 2010 Australian national psychosis survey used a two-phase design to draw a representative sample of adults aged 18–64 years with psychotic disorders in contact with public treatment services from an estimated resident population of 1 464 923 adults. This paper is based on data from 1642 participants with an International Classification of Diseases (ICD)-10 psychotic disorder. Its aim is to present estimates of treated prevalence and lifetime morbid risk of psychosis, and to describe the cognitive, physical health and substance use profiles of participants.

**Results:**

The 1-month treated prevalence of psychotic disorders was 3.10 cases per 1000 population aged 18–64 years, not accounting for people solely accessing primary care services; lifetime morbid risk was 3.45 per 1000. Mean premorbid intelligence quotient was approximately 0.5 s.d.s below the population mean; current cognitive ability (measured with a digit symbol coding task) was 1.6 s.d.s below the population mean. For both cognitive tests, higher scores were significantly associated with better independent functioning. The prevalence of the metabolic syndrome was high, affecting 60.8% of participants, and pervasive across diagnostic groups. Of the participants, two-thirds (65.9%) were current smokers, 47.4% were obese and 32.4% were sedentary. Of the participants, half (49.8%) had a lifetime history of alcohol abuse/dependence and 50.8% lifetime cannabis abuse/dependence.

**Conclusions:**

Our findings highlight the need for comprehensive, integrative models of recovery to maximize the potential for good health and quality of life for people with psychotic illness.

## Introduction

Psychotic illnesses comprise a heterogeneous group of disorders including schizophrenia, schizo-affective disorder, bipolar disorder with psychotic features, depression with psychosis and delusional disorders. Clinical onset of psychotic disorders occurs most often in late adolescence/early adulthood. These disorders are generally associated with persistent, or recurrent, and often disabling symptoms, and contribute substantially to the overall burden of years lived with disability (Vos *et al.* 2012). Recovery (symptom remission/reduction and functional improvement; Leucht & Lasser, 2006) is possible. However, improving outcome and quality of life for people with psychosis requires more than amelioration of symptoms. Cognition is impaired in a significant proportion of people with schizophrenia and is a critical determinant of poor functional outcome (Green *et al.* 2000; Gold, 2004). Cognitive impairment may precede illness onset (Reichenberg *et al.* 2005; Khandaker *et al.* 2011), and intelligence quotient (IQ) tends to decline over the course of illness (Woodberry *et al.* 2008). It is also well established that physical morbidity, especially cardiometabolic disease, and premature mortality are elevated in this group (Lawrence *et al.* 2001; Saha *et al.* 2007). In schizophrenia, life expectancy is reduced by 18.7 years for men and 16.3 years for women, compared with the general population (Laursen, 2011), and the gap is widening (Saha *et al.* 2007; Lawrence *et al.* 2013; Nielsen *et al.* 2013). Diseases of the circulatory system influence life expectancy more than death from external causes (Laursen, 2011). Antipsychotic medication is likely to contribute to high rates of cardiometabolic disorders, both directly, as well as mediated through weight gain side effects (De Hert *et al.* 2011). However, metabolic disturbance was observed in severe mental illness well before the introduction of antipsychotic medication (Maudsley, 1879), pointing to other key causal factors including modifiable life-style risks (obesity, smoking, substance abuse, low levels of physical activity and poor nutrition). General population studies also report an association between metabolic disturbance and cognitive dysfunction (Brands *et al.* 2005; Gao *et al.* 2008), predominantly in older samples with diabetes. However, few studies have examined this relationship in people with psychosis. Some (Dickinson *et al.* 2008; Lindenmayer *et al.* 2012; Han *et al.* 2013), but not all (Meyer *et al.* 2005), studies support the association, but the direction of causality in a disorder where cognition may be impaired early in its course remains indeterminate. From a positive perspective, there is good evidence of the effectiveness of cognitive remediation and other non-pharmacological interventions in improving specific outcomes in psychotic disorders, including cognitive function (Wykes *et al.* 2011) and physical health (Verhaeghe *et al.* 2011; Daumit *et al.* 2013).

The prevalence of common mental disorders is well documented (Kessler *et al.* 2007). However, few studies (Perälä *et al.* 2007; Kodesh *et al.* 2012) have reported estimates for psychotic disorders other than schizophrenia (Saha *et al.* 2005), and most do not include co-morbidities. In addition to prevalence estimates of specific psychotic disorders and co-morbidities, we also need simultaneously collected descriptive data on disability and social circumstances. These data are critical to mapping the extent of burden experienced across psychotic disorders, as well as identifying correlates of good outcome, in order to inform policy development and service planning.

With these data deficits in mind, the aims of the second Australian national survey of psychosis in 2010 (Survey of High Impact Psychosis; SHIP) were to estimate treated prevalence of psychosis for people aged 18–64 years in contact with public mental health services, including services provided through publicly funded non-government organizations (NGOs), and to describe, for these individuals, their mental and physical health, cognitive and other functioning, substance use, and personal, social and living circumstances. This paper reports, for psychosis overall, and for its component disorders, estimated 1-month treated prevalence and lifetime morbid risk (LMR), and rates of co-existing phenomena, focusing on cognition, physical ill-health and substance abuse. In addition, we take advantage of this large, unbiased sample of individuals in contact with treatment services to (i) examine whether those with greater cognitive impairment have poorer functioning and are at increased risk of the metabolic syndrome, and (ii) calculate the independent contribution of modifiable life-style risk factors to cardiometabolic disease.

## Method

### Population coverage

The 2010 Australian psychosis survey took place at seven catchment sites in five Australian states, covering an estimated resident population aged 18–64 years of 1 464 923 people, approximately 10% of Australians in that age range. The study sample comprised people aged 18–64 years, resident in the catchment sites and in contact with designated public mental health services (in-patient, out-patient, ambulatory and community mental health) and NGOs supporting people with mental illness. Detailed catchment profiles are available (Morgan *et al.* 2011). The census of people with psychosis was in March 2010. Interviews were conducted between April and December 2010.

### Design

A two-phase design was employed (Pickles *et al.* 1995). In phase 1, a psychosis screener (Jablensky *et al.* 1999, 2000) was used to identify individuals likely to meet diagnostic criteria. In addition to census-month enumeration, administrative records were examined in order to identify individuals with psychosis who were in contact with public mental health services in the 11 months prior to census but not in the census month. There were 7955 people who screened positive for psychosis who met eligibility criteria. In phase 2, 1825 people who were screen-positive in phase 1, and 164 who were screen-negative, were randomly selected, stratified by site and age group (18–34 years; 35–64 years) and interviewed. Data from the screen-negative group enabled estimation of prevalence without assuming that the psychosis screen had perfect sensitivity. The design and methodology have been described in full elsewhere (Morgan *et al.* 2011, 2012).

A 1-month treated prevalence was estimated per 1000 population aged 18–64 years by age strata and sex using sampling weights derived from phase 1 to phase 2 (Alonzo *et al.* 2003) and by expressing estimated numbers of persons in the screened population meeting diagnostic criteria as a proportion of the corresponding at-risk resident population of the catchment areas. LMR was estimated per 1000 population aged 18–45 years by sex using Weinberg's abridged method (Jablensky *et al.* 2011) (see online Supplementary Methods).

In the present study, descriptive data are for 1642 participants who were screen-positive for psychosis in phase 1 and who met full International Classification of Diseases (ICD)-10 criteria for a psychotic disorder in phase 2. Weighted estimates (see online Supplementary Methods) have been used.

#### Key assessments

##### Diagnostic classification

This was made using the Diagnostic Interview for Psychosis (DIP) (Castle *et al.* 2006). The DIP contains interview questions and probes, including items from the World Health Organization Schedules for Clinical Assessment in Neuropsychiatry (Wing *et al.* 1990) mapped onto the 90 diagnostic items of the Operational Criteria Checklist for Psychotic and Affective Illness (OPCRIT) (McGuffin *et al.* 1991). A computer algorithm provides diagnostic classification in accordance with ICD-10 and Diagnostic and Statistical Manual of Mental Disorders, fourth edition (DSM-IV) criteria on the basis of the DIP ratings, thus reducing subjective bias in the interpretation of symptoms and signs. Inter-rater reliability was good (see online Supplementary Methods).

##### Physical health assessment

Physical health assessment including blood pressure, height, weight and waist circumference, was undertaken by trained staff following standardized procedures and using identical equipment (see online Supplementary Methods). Participants provided fasting venous blood samples for assays of plasma glucose, triglycerides, high-density lipoprotein cholesterol and total cholesterol concentrations; standard methods in accredited pathology laboratories were employed. The World Health Organization body mass index reference range (World Health Organization, 2000) was used to classify obesity. Physical activity in the past 7 days was rated using the International Physical Activity Questionnaire (Craig *et al.* 2003) and classified according to Australian Bureau of Statistics categories (Australian Bureau of Statistics, 2008). Harmonized criteria (Alberti *et al.* 2009) were used to determine the metabolic syndrome.

##### Cognitive assessment

This involved two brief tests: the National Adult Reading Test-Revised (NART-R; Nelson & Willison, 1991) and the Digit Symbol Coding Test (DSCT) from the Repeatable Battery for the Assessment of Neuropsychological Status (Randolph, 1998). These tests provide reliable indices of cognitive ability prior to illness onset (NART-R) and currently (DSCT), and have been used extensively in previous studies of schizophrenia (Randolph, 1998; Smith *et al.* 1998; Crawford *et al.* 2001; Wilk *et al.* 2002; Dickinson *et al.* 2007). The NART-R Full-Scale IQ score was used to estimate premorbid IQ. The stability of NART-estimated intelligence over the long-term course of schizophrenia has been demonstrated (Morrison *et al.* 2000). The DSCT assesses cognitive processing efficiency. It requires the coordination and speeded performance of a range of simpler skills including: visual scanning, relational memory and motor ability. Lower coding scores signify relatively poorer performance, that is, greater information processing inefficiency. The DSCT is a robust indicator both of the presence and risk of illness (Dickinson *et al.* 2007) and functional outcome in schizophrenia (Gold *et al.* 2002).

##### Course of illness

Course of illness (single episode; multiple episodes of acute illness with partial/good recovery; continuous, chronic with/without deterioration) was assessed and rated by the interviewers who based their ratings on participant responses throughout the course of the interview.

##### Independent functioning

In the 4 weeks prior to interview, independent functioning was assessed by interviewers using the Multidimensional Scale of Independent Functioning (MSIF; Jaeger *et al.* 2003). Here we report on the Overall Global Independent Functioning scale which assessed functioning across occupational, educational and residential domains, correcting role functioning for degree of role responsibility, level of support provided, and actual performance: a score of 1 indicates functioning equivalent to community norms and 7 indicates total disability. The MSIF has been validated on samples of people with schizophrenia (Jaeger *et al.* 2003) and bipolar disorder (Berns *et al.* 2007) and has been used to assess the relationship between cognitive function and real-world outcomes in schizophrenia (Heinrichs *et al.* 2010). Since the MSIF does not measure sociability (‘social drive’), this was rated independently for the past year by the interviewers on the basis of multiple items in the interview tapping into this domain.

### Data analysis

Weighted analysis was done in IBM SPSS Modules for Complex Samples 21 (IBM, USA), which takes into account survey design and sampling weights. For the most part, population estimates are presented as percentages or means, with 95% confidence intervals (CIs). Conservatively, comparisons were considered statistically significant when CIs did not overlap (Julious, 2004). The SPSS Complex Samples General Linear Model was used to examine the association between cognition and cardiometabolic profile/risk factors. Complex Samples Logistic Regression was used to model univariate and multivariate relationships between the metabolic syndrome and life-style risk factors. s.d.s of means were calculated using Stata/IC 12.0 survey commands (StataCorp LP, USA).

## Results

### The 1-month treated prevalence of psychotic disorders and lifetime estimate of morbid risk

The 1-month treated prevalence of psychotic disorders in public mental health services was 3.10 cases per 1000 population aged 18–64 years (Table 1). Schizophrenia/schizo-affective disorder was the most prevalent diagnosis, and twice as prevalent in males as females. Bipolar disorder with psychosis was the next most frequent diagnosis, occurring substantially less frequently than schizophrenia/schizo-affective disorder and at comparable rates in males and females. The prevalence of depressive psychosis and delusional disorders was relatively low. LMR rates (Table 1) were somewhat higher than prevalence estimates but with comparable patterns of occurrence and sex differences.

**Table 1.** Estimated 1-month treated prevalence and lifetime morbid risk of individuals in contact with public treatment services and meeting criteria for ICD psychosis diagnoses (95% confidence intervals)

	Schizophrenia/schizo-affective disorder	Bipolar disorder with psychosis	Depressive psychosis	Delusional and other non-organic psychoses	All psychoses
Census, *n*	4928	1395	368	421	7112
Estimated 1-month treated prevalence (per 1000 population aged 18–64 years) by age strata and sex					
Age strata					
18–34 years	2.27 (2.13–2.42)	0.57 (0.35–0.79)	0.08 (0.04–0.13)	0.22 (0.13–0.30)	3.17 (2.95–3.38)
35–64 years	2.02 (1.83–2.21)	0.75 (0.61–0.88)	0.19 (0.12–0.26)	0.10 (0.05–0.14)	3.07 (2.88–3.25)
Sex					
Male	2.89 (2.68–3.10)	0.56 (0.43–0.70)	0.11 (0.05–0.17)	0.19 (0.12–0.25)	3.77 (3.54–4.01)
Female	1.35 (1.13–1.57)	0.79 (0.59–0.99)	0.19 (0.11–0.26)	0.10 (0.04–0.15)	2.44 (2.14–2.73)
Total	2.12 (1.99–2.25)	0.68 (0.56–0.79)	0.15 (0.10–0.20)	0.14 (0.10–0.18)	3.10 (2.97–3.24)
Estimated lifetime morbid risk (per 1000 population aged 18–45 years) by sex					
Sex					
Male	3.31 (3.07–3.55)	0.64 (0.49–0.80)	0.12 (0.05–0.20)	0.21 (0.14–0.29)	4.29 (4.03–4.56)
Female	1.48 (1.23–1.72)	0.86 (0.64–1.08)	0.21 (0.12–0.29)	0.11 (0.05–0.17)	2.65 (2.33–2.97)
Total	2.37 (2.23–2.52)	0.76 (0.62–0.89)	0.17 (0.11–0.22)	0.16 (0.11–0.21)	3.45 (3.30–3.61)

ICD, International Classification of Diseases.

### The 12-month interviewed sample

The number of people meeting ICD-10 criteria for a psychotic disorder was 1642 (weighted *n* = 7112). Online Supplementary Tables S1–S14 include descriptive data on the interviewed sample by diagnostic group. The proportion of males was 61.1% (range 39.7–70.4% across the five diagnostic groups). The mean age at onset was 24 years (range 23–25 years) and the mean age at interview was 39 years (range 39–42 years). Mean duration of illness was 16 years (range 15–17 years) (see online Supplementary Tables S1 and S3).

### Course of illness and functioning

The majority of participants (61.6% in total, range 54.8–77.6% across the diagnostic groups) were rated as having multiple episodes of illness with good or partial recovery between episodes (online Supplementary Table S3). For one-third (32.5%), however, course of illness was continuous. The proportion was highest for schizophrenia (38.8%) and lowest for bipolar disorder with psychosis (18.9%); this difference was significant (*p*<0.000). The mean score for global independent functioning was 3.5. The highest mean score (indicating poorest performance) was for schizophrenia and the lowest was for depressive psychosis (online Supplementary Table S9). Overall, one-quarter (23.5%) of participants were rated as normal or very mildly disabled relative to community norms with respect to independent functioning while almost another quarter (22.2%) were assessed as significantly, extremely or totally disabled. In addition, almost two-thirds (65.1%) of participants were rated as having obvious or severe dysfunction in social drive. This figure was highest for depressive psychosis (79.4%) and lowest for bipolar disorder with psychosis (58.1%), a significant difference.

### Cognitive impairment

The proportions of participants with valid NART-R and DSCT data were 84.7% and 88.7%, respectively. Mean estimated premorbid IQ (NART-R), 98.0 (s.d. = 11.3), was approximately 0.5 s.d.s below the population mean (Nelson & Willison, 1991) of 107.4 (s.d. = 17.1) (online Supplementary Table S5). Current cognitive ability (DSCT) was markedly impaired: participants had a mean score of 38.3 (s.d. = 10.6), which is 1.6 s.d.s below the population mean (Australian Schizophrenia Research Bank, 2011) of 54.2 (s.d. = 9.8). DSCT raw scores were also examined as a function of age group (Table 2). In both the survey and Australian normative samples, younger age groups performed better than older age groups. However, mean scores for the youngest survey participants were significantly lower than those for all normative age groups, including the oldest. Each diagnostic group scored below the population mean on the NART-R, albeit within 1 s.d., ranging from 0.4 s.d.s (bipolar disorder with psychosis) to 0.7 s.d.s (delusional disorders) below the norm (online Supplementary Table S5). Participants with schizophrenia scored significantly lower than those with bipolar disorder. For the DSCT, each diagnostic group scored over 1 s.d. below the norm, ranging from 1.2 s.d.s (depressive psychosis) to 1.7 s.d.s (schizophrenia, schizo-affective disorder and delusional disorders) below the norm (online Supplementary Table S5). Participants with schizophrenia scored significantly lower than those with bipolar disorder or depressive psychosis. Finally, higher NART-R and DSCT scores were both significantly associated with lower (better) scores on global independent functioning [general linear model regression estimates of −1.2 (95% CI −1.7 to −0.7) and −2.7 (95% CI −3.2 to −2.3), respectively].

**Table 2.** Current cognitive function^a^ by age group

Age group, years	2010 Australian psychosis survey	Australian population norms^b^
	Mean (s.d., 95% CI)	Mean (s.d.)
18–24	41.7 (11.0, 39.1–44.2)	59.9 (9.6)
25–34	40.2 (9.9, 39.1–41.3)	59.7 (9.1)
35–44	38.7 (9.8, 37.6–39.9)	55.5 (8.3)
45–54	34.7 (10.4, 33.0–36.4)	51.8 (8.4)
55–64	33.6 (11.2, 31.6–35.6)	48.1 (8.9)

s.d., Standard deviation; CI, confidence interval.

a Based on speed of information processing using the Digit Symbol Coding Test (Randolph, 1998).

b Australian Schizophrenia Research Bank (2011).

### Cardiometabolic disease and its risk factors

Of participants who provided fasting blood samples (*n* = 1155, 70.3% of the total), 60.8% met harmonized criteria (Alberti *et al.* 2009) for the metabolic syndrome. The proportions meeting thresholds for each component of the metabolic syndrome, or in treatment for the component condition, were: increased abdominal adiposity, 84.2%; reduced high-density lipoproteins, 58.1%; elevated triglycerides, 55.5%; elevated blood pressure, 54.4%; and elevated glucose, 35.3% (online Supplementary Table S4). The prevalence of the metabolic syndrome and its component criteria was comparable across the diagnostic groups.

Many participants had potentially modifiable life-style risk factors for cardiometabolic disease (online Supplementary Table S4). Two-thirds (65.9%) were current smokers (males 70.3%; females 59.0%), consuming 21 cigarettes per day on average, with 40.1% of the total meeting criteria for high or very high nicotine dependence (Fagerström Test for Nicotine Dependence; Heatherton *et al.* 1991). Of the participants, one-third (32.4%) was assessed as being sedentary in the last 7 days, with a further 63.6% recording low levels of activity. As many as 70.6% had one or fewer serves of fruit per day, while 48.1% had one or fewer serves of vegetables per day. In addition to 84.2% meeting the abdominal adiposity threshold for the metabolic syndrome, 47.4% had a body mass index in the obese range. Almost two-fifths (38.8%) of participants reported weight gain associated with medication use: the mean reported gain over the previous 6 months was 9.5 kg.

Table 3 shows the relationship between cognition and the metabolic syndrome. Lower current cognitive performance (DSCT) was significantly associated with having the metabolic syndrome and with meeting threshold levels for each of its criteria. By contrast, there were no significant associations between premorbid IQ (NART-R) and the same measures. Examining life-style risk factors, lower current cognitive performance was significantly associated with body mass index, smoking and physical activity level, but not with fruit and vegetable consumption. Lower premorbid IQ was significantly associated with smoking only.

**Table 3.** Cognitive function and metabolic parameters

	Premorbid IQ^a^		Current cognitive function^b^	
	Mean	*F*, *p*	Mean	*F*, *p*
Metabolic syndrome^c^ and threshold criteria				
Met criteria for the metabolic syndrome^c^				
No	98.5	*F* = 0.1, *p* = 0.717	40.2	*F* = 21.3, *p* = 0.000
Yes	98.2		36.9	
Abdominal obesity				
No	97.3	*F* = 1.1, *p* = 0.289	39.8	*F* = 4.3, *p* = 0.039
Yes	98.2		38.0	
Reduced high-density lipoprotein levels				
No	99.3	*F* = 3.8, *p* = 0.052	39.5	*F* = 9.5, *p* = 0.002
Yes	97.8		37.3	
Elevated triglyceride levels				
No	98.3	*F* = 0.0, *p* = 0.903	39.6	*F* = 13.9, *p* = 0.000
Yes	98.4		37.0	
Elevated glucose levels				
No	98.2	*F* = 0.1, *p* = 0.703	39.2	*F* = 12.6, *p* = 0.000
Yes	98.5		36.7	
Elevated blood pressure				
No	98.3	*F* = 0.2, *p* = 0.694	39.7	*F* = 20.6, *p* = 0.000
Yes	98.0		37.0	
Life-style risk factors				
Current smoker				
No	100.1	*F* = 20.2, *p* = 0.000	39.3	*F* = 6.5, *p* = 0.011
Yes	96.9		37.6	
Body mass index				
Underweight/normal	97.7	*F* = 0.4, *p* = 0.690	40.0	*F* = 4.4, *p* = 0.012
Overweight	98.5		38.2	
Obese	97.9		37.6	
Level of physical activity				
Very low	98.1	*F* = 0.2, *p* = 0.903	36.8	*F* = 3.1, *p* = 0.025
Low	98.0		38.8	
Moderate	97.6		39.0	
High	102.2		39.3	
One or fewer serves of vegetables per day				
No	97.6	*F* = 1.2, *p* = 0.267	38.2	*F* = 0.1, *p* = 0.802
Yes	98.4		38.3	
One or fewer serves of fruit per day				
No	97.8	*F* = 1.6, *p* = 0.212	38.3	*F* = 0.2, *p* = 0.688
Yes	98.7		38.1	

IQ, Intelligence quotient.

a Measured using the National Adult Reading Test-Revised (Nelson & Willison, 1991).

b Based on speed of information processing using the Digit Symbol Coding Test (Randolph, 1998).

c Based on harmonized criteria (Alberti *et al*. 2009).

Further analysis was undertaken to assess the independent contribution of modifiable life-style risk factors to the metabolic syndrome. In univariate analyses, current smoking, body mass index and activity level, but not vegetable or fruit consumption, were significant predictors. In the unadjusted multivariate model, current smoking and body mass index remained significant. These variables were retained and the model was adjusted for sex, age, illness duration and socio-economic status. Further adjustment was made for potential confounders, first separately for medication use, diagnostic classification and current cognitive ability, and then for the three potential confounders combined. The results were virtually unchanged in all models and none of the added variables was significant (see Table 4).

**Table 4.** Life-style risk factors and the metabolic syndrome

	Univariate unadjusted odds ratio (95% CI)	Multivariate unadjusted odds ratio (95% CI)	Multivariate adjusted^a^ odds ratio (95% CI)	Multivariate adjusted^b^ odds ratio (95% CI)
Life-style risk factors				
Currently a smoker				
Yes	1.3 (1.0–1.7)*	1.9 (1.4–2.6)*	2.2 (1.6–3.0)*	2.3 (1.6–3.2)*
No	Reference	Reference	Reference	Reference
Body mass index				
Underweight or normal	Reference	Reference	Reference	Reference
Overweight	4.8 (3.2–7.2)*	5.3 (3.5–8.0)*	5.3 (3.5–8.2)*	4.9 (3.1–7.8)*
Obese	13.9 (9.4–20.7)*	16.6 (11.0–25.2)*	17.9 (11.6–27.7)*	16.8 (10.4–27.0)*
Activity level				
Very low (sedentary)	2.5 (1.3–5.1)*	–	–	–
Low	1.8 (0.9–3.5)	–	–	–
Moderate or high	Reference	–	–	–
One or fewer serves of vegetables per day				
Yes	1.1 (0.9–1.4)	–	–	–
No	Reference	–	–	–
One or fewer serves of fruit per day				
Yes	0.9 (0.7–1.2)	–	–	–
No	Reference	–	–	–

CI, Confidence interval.

a Adjusted for sex, age, illness duration and socio-economic status.

b Adjusted for sex, age, illness duration and socio-economic status, and diagnosis, cognition and medication.

* *p* < 0.05.

### Co-morbid substance abuse

Almost half (49.8%) of the participants had a lifetime history of alcohol abuse/dependence (males 57.2%; females 38.0%). The proportion was highest for delusional disorder (54.1%) and lowest for schizo-affective disorder (46.5%). Based on consumption over the past year, 17.0% of the total sample met criteria for hazardous drinking and 12.9% for harmful or dependent drinking (Alcohol Use Disorder Identification Test) (Babor *et al.* 2001). The proportion with current harmful or dependent drinking was highest for depressive psychosis (21.9%) and lowest for schizophrenia (11.3%). In addition, 50.8% had a lifetime history of cannabis abuse/dependence (males 60.2%; females 36.2%). The proportion was highest for schizophrenia (54.1%) and lowest for depressive psychosis (40.5%). Over the past year, 30.8% of all participants had been using cannabis; 11.9% were using it daily. People with schizophrenia were least likely to be using cannabis daily (10.0%) and those with schizo-affective disorder were most likely (15.6%) (see online Supplementary Tables S7 and S8).

## Discussion

The 1-month treated prevalence estimate for psychosis in Australia is 3.10 per 1000 population aged 18–64 years, while the LMR is estimated to be 3.45 per 1000 population aged 18–45 years. These figures do not take into account people solely accessing primary care or private mental health services for their disorder, or not in contact with any treatment service. Mindful of the more restricted age range of the LMR, the Australian LMR estimate is relatively low compared with international estimates (median per 1000 = 7.20, interquartile range = 4.7–17.20) (Saha *et al.* 2005). Estimates from a nationally representative sample in Finland have reported a lifetime prevalence for psychotic disorders, a value usually lower than LMR, as high as 34.8 per 1000 (Perälä *et al.* 2007). In keeping with the relatively low prevalence of psychotic disorders, the estimates for the subtypes are also low compared with published estimates. In particular, the estimated LMR for bipolar disorder with psychosis was only 0.76 per 1000. Since our survey was specifically designed to capture psychotic disorders rather than all mood disorders, the estimates apply to affective psychoses only, and represent a lower boundary.

In keeping with previous findings (Reichenberg *et al.* 2005; Khandaker *et al.* 2011), we found cognitive impairment in people with psychosis that preceded illness onset. The mean estimated premorbid IQ for the survey participants was approximately 0.5 s.d.s below the population mean, similar to results by Morrison *et al.* (2000) of 96.9 (s.d. = 14, range 69–124), and to s.d.s of 0.4 and 0.5 below the population mean reported in two recent reviews (Woodberry *et al.* 2008; Khandaker *et al.* 2011). Our data also support the evidence of further cognitive impairment after illness onset (with DSCT scores slightly more than 1.5 s.d.s below the population mean) and of associated functional deficits, with both current and premorbid cognitive impairment related to poorer independent functioning. Finally, we found that current cognitive impairment, as measured by a speed of information processing task, was associated with cardiometabolic measures and life-style risk factors for cardiometabolic disease although we were unable to explore directionality in our cross-sectional data. The specific association with speed of information processing has been observed in general population studies (Brands *et al.* 2005), as well as schizophrenia studies (Dickinson *et al.* 2008; Lindenmayer *et al.* 2012). The pathophysiology underlying the association between metabolic dysregulation and cognition remains unclear. It is likely to involve the joint effects of hyperglycaemia and hyperinsulinaemia increasing the risk of cerebral vascular abnormalities and structural brain changes either directly or mediated by mechanisms such as altered synaptic plasticity, oxidative stress, advanced glycation end-products and inflammation (Biessels *et al.* 2006; Panza *et al.* 2010; Jones, 2012). While an association has been reported between metabolic disturbance and cognitive deficits, including speed of processing deficits, in schizophrenia (Meyer *et al.* 2005; Lindenmayer *et al.* 2012), further investigation of possible bidirectionality is warranted in psychotic illness where cognitive impairment antedates the onset of metabolic disorders. In addition, cognitive impairment may exacerbate vulnerability for the metabolic syndrome via a variety of mechanisms including reduced responsiveness to public health measures targeting life-style risk factors.

Some 60.8% of participants met criteria for the metabolic syndrome, significantly higher than the prevalence of 32.5% for schizophrenia reported in a recent systematic review (Mitchell *et al.* 2013). Our prevalence is similar to the 54% reported for a clinical sample using more conservative International Diabetes Federation criteria (International Diabetes Federation, 2006) in one of a very few Australian studies (John *et al.* 2009), although another study found a 68% rate in a chronic, predominantly hospitalised, sample (Tirupati & Chua, 2007). Our data underline the critical role of modifiable life-style risk factors for cardiometabolic disease, especially body mass index and smoking. It has been proposed that smoking cessation would produce a 75% reduction in high/very high risk of cardiovascular disease in people with schizophrenia (Bobes *et al.* 2010). Of concern, based on comparable subsamples from the 2010 and 1997–1998 national psychosis surveys, smoking rates had not changed over 13 years (Morgan *et al.* 2012). Cardiorespiratory fitness has also been associated with reduced all-cause mortality as well as mortality from cardiovascular disease (Wildgust & Beary, 2010) and there is growing evidence for the mental health benefits of physical exercise (Erickson *et al.* 2011). However, 32.4% of participants were sedentary and a further 63.6% recorded low levels of activity. Proportions for the Australian population in the same age range are 18.0% and 54.0%, respectively (Morgan *et al.* 2011, 2012). We did not find that diet was related to the metabolic syndrome. A recent review of this area confirms that people with schizophrenia have a poor diet, but notes that evidence linking diet to metabolic abnormalities in this population is equivocal and further research is needed (Dipasquale *et al.* 2013). Intervention trials support the effectiveness of life-style modifications in this population (Verhaeghe *et al.* 2011; Daumit *et al.* 2013). Nonetheless, we need to know more about how best to promote, implement and support life-style changes in the community context, especially when the majority of our participants experience impaired social drive.

People with severe mental illness also face inequalities in physical health service delivery (Lawrence & Kisely, 2010). We have previously reported that the proportion of survey participants having a blood test in the previous year had dropped markedly since the 1997–1998 survey, from 83.1% to 64.8%, while the proportion having had a physical examination had also dropped – from 79.6% to 66.1% (Morgan *et al.* 2012). Moreover, only half (51.7%) of 2010 survey participants with hypertension were on medication for their condition; the figures for diabetes/hyperglycaemia and hypercholesterolaemia were even lower, at 39.8% and 39.4%, respectively (Galletly *et al.* 2012). These findings support the need for improved primary care interventions for this population, and the integration of general medical and mental health treatment (Jerrell *et al.* 2012).

Across the diagnostic groups, we observed some expected differences. Diagnostic groups differed in terms of course of disorder, symptom profiles, social drive (but not global independent performance) and psychotropic medication use. The finding of significantly lower premorbid IQ in schizophrenia compared with bipolar disorder with psychosis is consistent with the literature (Zammit *et al.* 2004). We also found that current cognitive ability, measured by a speed of information processing task, was significantly lower in schizophrenia compared with both bipolar disorder with psychosis and depressive psychosis. However, poor physical health was pervasive, as evidenced by high rates of the metabolic syndrome, of any of the threshold criteria for the metabolic syndrome or its modifiable life-style risk factors, and in the high prevalence of lifetime alcohol, cannabis and other substance abuse/dependence across all diagnostic groups.

### Strengths and limitations

The 2010 Australian national survey of psychosis is one of the most comprehensive worldwide. Many of its 1500 data items have not previously been assessed contemporaneously and in such depth in a large, representative, national sample. The inclusion of the range of psychotic disorders in a sample of this size has enabled an assessment of the specificity of findings across the psychosis spectrum. The epidemiological sampling design ensures that findings are generalizable to adults in contact with public mental health treatment services in developed countries in similar public treatment service settings. We did not enumerate those solely in treatment in general medical practices or private psychiatric/psychological settings, or homeless people not in contact with any mental health services. We estimate that 1-month prevalence would have risen from 3.1 to 5.3 per 1000 population, if we had accounted for these and those in the NGO sector (Morgan *et al.* 2012). In addition, we did not enumerate those in prison or forensic mental health services. Underestimation of prevalence may have occurred if people with psychosis were missed during screening, or if refusal/inability to participate was associated with a higher likelihood of illness exacerbation. However, comparison of screening data, including symptom profiles, between interviewed participants and those selected for interview but not participating for any reason, indicated no systematic selection biases. The impact of normal sampling variation, errors in population estimates and diagnostic misclassification is likely to be either negligible or to result in underestimation of prevalence.

## Conclusions

People with psychosis continue to experience poor physical health, even though many of their risk factors are modifiable and despite public health campaigns aimed at these very risk factors. The World Health Organization has identified tobacco use, high blood pressure, overweight and obesity, physical inactivity, high blood glucose, high cholesterol, low fruit and vegetable intake, and alcohol use among the 10 top risks for mortality and disability in high-income countries (World Health Organization, 2009). Our survey found high rates of all eight risk factors among participants, with no significant differences between the diagnostic groups, highlighting urgent need for physical health interventions across the range of psychotic disorders. Moreover, the observed deficit in current cognitive function and its relationship with both physical health and global functioning suggests that cognitive remediation must be an integral component of intervention for people with psychotic illness, with important implications for workforce planning. At the same time, further investigation is warranted into who will have good mental and physical outcomes, and why. One-quarter of our participants had no or only mild impairment in global independent functioning, and two in five did not have the metabolic syndrome. Our data challenge services to establish comprehensive and integrative models of recovery in order to maximize the potential for good health and quality of life for all people with psychotic illness.

## Supplementary Material

Supplementary Material Supplementary information supplied by authors.

## Supplementary material

For supplementary material accompanying this paper, please visit http://dx.doi.org/10.1017/S0033291713002973.
