# Supplementary material for: Psychosis prevalence and physical, metabolic and cognitive co-morbidity: data from the second Australian national survey of psychosis
Source: Psychol Med. 2014 Jan 2;44(10):2163–76. doi: 10.1017/S0033291713002973 (PMC4045165; doi:10.1017/S0033291713002973)
Supplement: Supplementary Material — Supplementary information supplied by authors. [file S0033291713002973sup001.doc]

**Supplementary File: Methods and Tables**

**Psychosis prevalence and physical, metabolic and cognitive comorbidity. Data from the second Australian national survey of psychosis**

VA Morgan, JJ McGrath, A Jablensky, JC Badcock, A Waterreus, R Bush, V Carr, D Castle, M Cohen, C Galletly, C Harvey, B Hocking,

P McGorry, AL. Neil, S Saw, S Shah, HJ. Stain, A Mackinnon

CONTENTS

[Supplementary Methods 2](#__RefHeading___Toc364258000)

[Estimation of one-month treated prevalence 2](#__RefHeading___Toc364258001)

[Lifetime morbid risk 2](#__RefHeading___Toc364258002)

[Sample weights 2](#__RefHeading___Toc364258003)

[Participant response rates 2](#__RefHeading___Toc364258004)

[Inter-rater reliability 2](#__RefHeading___Toc364258005)

[Physical health assessments 3](#__RefHeading___Toc364258006)

[Physical health calculations 4](#__RefHeading___Toc364258007)

[Supplementary Tables 5](#__RefHeading___Toc364258008)

[Table S1. Sociodemographic profile 5](#__RefHeading___Toc364258009)

[Table S2. Marital and parenting status 6](#__RefHeading___Toc364258010)

[Table S3. Mental health profile 7](#__RefHeading___Toc364258011)

[Table S4. Physical health profile 8](#__RefHeading___Toc364258012)

[Table S5. Cognitive profile 9](#__RefHeading___Toc364258013)

[Table S6. Smoking 10](#__RefHeading___Toc364258014)

[Table S7. Alcohol use 11](#__RefHeading___Toc364258015)

[TableS 8. Substance use 12](#__RefHeading___Toc364258016)

[Table S9. Functioning, quality of life and social relationships 13](#__RefHeading___Toc364258017)

[Table S10. Victimisation and offending 14](#__RefHeading___Toc364258018)

[Table S11. Medication and medication side effects 15](#__RefHeading___Toc364258019)

[Table S12. Service utilisation 16](#__RefHeading___Toc364258020)

[Table S13. Visits to general practitioner 17](#__RefHeading___Toc364258021)

[Table S14. Physical examinations 18](#__RefHeading___Toc364258022)

[References 19](#__RefHeading___Toc364258023)

# Supplementary Methods

## Estimation of one-month treated prevalence

Population one-month treated prevalence was estimated using sampling weights derived from phase 1 to phase 2 and by expressing estimated numbers of persons in the screened population meeting diagnostic criteria as a proportion of the corresponding at-risk resident population of the catchment areas. Confidence intervals for these values were calculated by considering the resident population estimates to be fixed and expressing the upper and lower bounds of estimated numbers meeting diagnostic criteria. Diagnostic status was ascertained at interview and the proportion of screened people meeting criteria for an ICD-10 diagnosis of psychosis was estimated using appropriately weighted screen-positive and screen-negative subsamples. From this proportion, the number of people meeting diagnostic criteria in the census month was estimated for each site. Aggregate estimates in each age and sex stratum were obtained by combining the fraction of the estimated resident population of each catchment meeting diagnostic criteria. For estimates combining strata, adjustments were made to reflect the age and sex distributions of the Australian population aged 18-64 years. The statistical package Stata/IC version 12.1 was used for prevalence estimation.

## Lifetime morbid risk

Lifetime morbid risk (LMR) is the probability of a person developing a disorder during a specified period of their life or up to a specified age. It attempts to cover the entire lifetime of a birth cohort, both past and future, and includes those deceased at the time of the survey . We estimated LMR of psychotic disorder using Weinberg's abridged method . This involves adjusting the denominator of ratio of persons affected to population numbers according to the formula:

$$LMR=\frac{A}{B-(B_{0}+1/2B_{m})}$$

where A is the number of prevalent cases; B is the total catchment population or subpopulation; B_0_ is the number of persons who have not yet entered the risk period; and B_m_ is the number of persons within the risk period. Reflecting the spectrum of psychotic disorders, the period of risk was defined as 18 to 45 years. Catchment resident population estimates for persons outside the survey age range, which was 18-64 years, were not available so estimation was based on prorating those of the whole Australian population.

## Sample weights

Phase 2 estimates were calculated using sampling weights reflecting the number of screen-positive and screen-negative participants interviewed by site, sex and within each of two age strata (18-34 and 35-64 years). The estimates therefore reflect the census population as screened.

## Participant response rates

The response rate among 4,189 people selected and contacted for interview from those screen-positive for psychosis and randomised was 44%. A further 2,107 people had been randomly sampled for interview but were not asked to participate because (i) they could not be traced or had died (57%), (ii) case managers had assessed them as too unwell mentally or had not contact them about the survey (21%), or (iii) interviewers judged them to be too unwell to provide consent (22%). A flow chart of case enumeration has been published .

## Inter-rater reliability

Interviewers were predominantly mental health professionals trained to use the survey instruments and to take standardised physical measures. They received specialised training in administering and scoring the Diagnostic Interview for Psychosis (AJ) and cognitive tasks (JCB). Procedures were implemented to ensure quality and reliability. Inter-rater reliability was assessed in the course of field interviews (AJ, HS), with good agreement among interviewers (averaged pairwise agreement of 0.94 for ICD-10 diagnoses and intra-class correlation of 0.98 for the NART-R).

## Physical health assessments

Blood Sampling

- For analysis of total cholesterol, high density lipoprotein, triglyceride and plasma glucose levels.
- One authorised pathology service at each site collected, analysed and reported results.
- Participants were asked to fast from midnight the night before testing.
- Participants unable to fast undertook the test with the non fasting time recorded.
- 5.5mls blood were collected.
- Results were recorded in mmol/L to two decimal points

Waist circumference

- Perfect waist tape measures were provided to all interviewers.
- It is made of fiberglass fabric which is flexible and won't stretch like cotton fabric does.
- The tape was wrapped around the waist; the peg at the end was secured into the case, and cinched until snug.
- Waist circumference was measured as recommended in the NIH guidelines by locating the top of the hip bone (iliac crest). The tape measure was placed evenly around the abdomen at the level of this bone. The tape measure was snug without compressing the skin. The participant was asked to breathe out gently and the measurement was taken at end of a normal expiration.
- Measurements were recorded to the nearest centimetre.

Height

- Interviewers used wall mounted height measuring devices or standard tape measures attached to the wall for readings.
- Participants were asked to remove shoes. Each participant was positioned to be standing fully erect with heels, buttocks and shoulders resting lightly against the wall, in front of, the measurement device. Readings were taken to the nearest centimetre.

Weight

- All interviewers used scales provided: Propert maxi weigh glass electronic scales capacity 200kg model 3202
- Scales placed on a firm and even surface
- Scales turned on by pressing the centre of the glass platform with foot. The display showed 0.0.
- Participant’s shoes were removed.
- The participant was asked to stand on the scales making sure feet were placed evenly and standing still.
- Weight was recorded from digital display on scales and was recorded to the nearest kilogram.

Blood pressure

- All interviewers used blood pressure monitors provided: AND digital blood pressure monitor model UA-767 plus
- Ideally, the participant should not have consumed caffeine or smoked for at least 2 hours before BP was measured.
- Participants were seated and had been for at least 5-10 minutes before measurement.
- The appropriate cuff size was selected (medium or large). The selected arm (preferably left) was freed of constricting clothing so that the cuff could be wrapped around the upper arm without impediment.
- The cuff was wrapped around the upper arm about 2-3cms above the elbow with the airhose extended towards the hand and in the middle of the arm.
- The START button was pressed.
- If an appropriate pressure was not obtained the machine automatically inflated again.
- When completed the readings were displayed and recorded.

## Physical health calculations

Body Mass Index (BMI)

Body mass index was calculated from measured height and weight, using the following formula: weight in kilograms divided by height in meters squared. BMI values are grouped according to the list below which allows categories to be reported against the World Health Organization (WHO) guidelines .

- Underweight (BMI < 18.5)
- Normal (BMI 18.50–24.99)
- Overweight (BMI 25.00–29.99)
- Obese (BMI ≥ 30).

Physical Activity

The International Physical Activity Questionnaire was used to assess the amount of time participants spent in both vigorous and moderate exercise, and the amount of time they spent sitting on a typical weekday, during the last 7 days. The total time spent in various activities over the previous 7 days was classified according to criteria from the National Survey of Mental Health and Wellbeing into four levels of activity:

- Very low: less than 100 minutes (including no exercise)
- Low: 100 minutes to less than 1,600 minutes
- Moderate: 1,600–3,200 minutes, or more than 3,200 minutes, but less than 2 hours of vigorous exercise
- High: more than 3,200 minutes including 2 hours or more of vigorous exercise.

Metabolic Syndrome

Metabolic syndrome was defined using the harmonized criteria developed by the International Diabetes Federation Task Force on Epidemiology and Prevention and related expert organizations . These criteria for metabolic syndrome require three of the following five risk factors to make the diagnosis:

- Abdominal obesity (at-risk waist circumference): waist circumference ≥ 94 cm for men and ≥ 80 cm for women.
- At-risk diastolic and/or systolic blood pressure: systolic blood pressure ≥ 130 mmHg and/or diastolic pressure ≥ 85 mmHg.
- At-risk levels of fasting blood glucose: glucose ≥ 5.6 mmol/l.
- At-risk levels of fasting triglycerides: triglycerides ≥ 1.7 mmol/l.
- At-risk levels of fasting lipids: HDL-C < 1.0 mmol/l for men and < 1.3 mmol/l for women.

People receiving medications for hypertension, hyperlipidaemia or hyperglycaemia were considered to meet the relevant criterion.

# Supplementary Tables

| TABLE 1. SOCIODEMOGRAPHIC PROFILE | | | | | | |
| --- | --- | --- | --- | --- | --- | --- |
|  | Schizophrenia | Schizoaffective disorder | Bipolar disorder  with psychosis | Depressive  psychosis | Delusional and other nonorganic psychoses | Any psychosis |
|  | N=857 | N=293 | N=319 | N=81 | N=92 | N=1642 |
|  | % (95%CI) | % (95% CI) | % (95% CI) | % (95% CI) | % (95% CI) | % (95% CI) |
| **Age, sex, place of birth, language** |  |  |  |  |  |  |
| Aged 35-64 years | 59.8 (56.4-63.1) | 60.9 (55.0-66.5) | 72.0 (66.7-76.7) | 66.8 (55.5-76.4) | 60.6 (49.8-70.5) | 62.8 (60.3-65.2) |
| Age at interview (mean years; 95% CI) | 39 (38-39) | 39 (38-41) | 42 (40-43) | 41 (38-44) | 39 (37-42) | 39 (39-40) |
| Male | 70.4 (67.1-73.6) | 53.6 (47.5-59.5) | 44.5 (38.8-50.3) | 39.7 (29.1-51.3) | 72.0 (61.4-80.7) | 61.1 (58.6-63.6) |
| Born in Australia | 83.6 (80.9-86.0) | 83.4 (78.5-87.4) | 81.9 (77.1-85.8) | 79.8 (69.6-87.2) | 86.4 (77.6-92.1) | 83.2 (81.3-85.0) |
| First language is not English | 11.3 (9.3-13.8) | 9.4 (6.4-13.6) | 7.9 (5.3- 11.7) | 6.4 (2.9-13.6) | 9.9 (5.2-18.2) | 10.0 (8.6-11.7) |
| **Income and education** |  |  |  |  |  |  |
| Private health insurance | 13.2 (11.0-15.7) | 10.9 (7.7-15.0) | 17.8 (13.8-22.7) | 22.9 (14.4-34.4) | 15.1 (8.9-24.4) | 14.3 (12.7-16.2) |
| Main income source: government pension, past year | 88.9 (86.5-90.9) | 86.8 (82.1-90.3) | 78.1 (72.9-82.5) | 76.2 (64.9-84.8) | 85.1 (75.1-91.5) | 85.5 (83.6-87.2) |
| **Education** |  |  |  |  |  |  |
| Completed Year 12 education | 30.2 (27.1-33.6) | 31.3 (26.0-37.2) | 39.4 (33.9-45.2) | 29.2 (19.9-40.6) | 20.8 (13.5-30.8) | 31.6 (29.3-34.0) |
| Enrolled in formal studies (past year) | 18.0 (15.5-20.9) | 22.8 (18.2-28.2) | 20.5 (16.1-25.7) | 26.0 (17.3-37.1) | 17.0 (10.0-27.2) | 19.6 (17.7-21.8) |
| **Employment** |  |  |  |  |  |  |
| In paid employment (past year) | 31.5 (28.3-34.9) | 26.2 (21.3-31.8) | 39.7 (34.2-45.5) | 38.5 (27.9-50.3) | 35.3 (25.6-46.4) | 32.8 (30.5-35.3) |
| In paid employment (past 7 days) | 20.7 (18.0-23.8) | 17.8 (13.7-22.8) | 24.9 (20.3-30.2) | 24.7 (16.0-36.2) | 21.0 (13.4-31.4) | 21.3 (19.3-23.5) |
| *If working:* |  |  |  |  |  |  |
| Competitive employment | 68.6 (62.2-74.4) | 73.8 (62.0-82.9) | 78.0 (68.8-85.0) | 73.5 (52.2-87.6) | 58.6 (39.6-75.2) | 71.2 (66.7-75.2) |
| Part-time employment | 75.1 (69.4-80.1) | 64.7 (53.2-74.7) | 69.3 (60.5-76.9) | 56.4 (37.2-73.9) | 63.2 (44.2-78.9) | 70.5 (66.3-74.3) |
| Hours per week (mean hours; 95%CI) | 21 (19-23) | 25 (21-30) | 25 (22-27) | 25 (19-31) | 23 (16-29) | 23 (21-24) |
| **Accommodation at time of interview** |  |  |  |  |  |  |
| Own or rented home/unit | 55.8 (52.2-59.2) | 68.3 (62.4-73.6) | 68.3 (62.7-73.4) | 77.7 (66.7-85.9) | 58.3(47.2-68.6) | 61.6 (59.0-64.0) |
| Family home | 20.9 (18.2 -23.8) | 16.3 (12.3 -21.2) | 18.2 (14.2 -23.0) | 11.6 (6.0 -21.5) | 12.3 (7.0 -20.8) | 18.6 (16.7 -20.6) |
| Supported group housing | 14.0 (11.6-16.7) | 8.9 (6.1-13.0) | 7.1 (4.5-10.9) | 5.0 (1.8-13.2) | 22.6 (14.4-33.7) | 11.8 (10.2-13.7) |
| Homeless - primary, secondary or tertiary***** | 6.2 (4.7-8.1) | 3.8 (2.1-6.7) | 4.1 (2.4-7.0) | 3.9 (1.4-10.1) | 3.8 (1.2-11.4) | 5.1 (4.2-6.4) |
| **Homelessness* (past year)** |  |  |  |  |  |  |
| Any periods of homelessness | 11.8 (9.8-14.2) | 11.6 (8.3-16.1) | 12.1 (8.7-16.5) | 14.8 (8.3-25.2) | 17.0 (10.2-26.9) | 12.3 (10.7-14.0) |
| No. of days of homelessness (mean days; 95%CI) | 176 (147-205) | 124 (82-167) | 143 (95- 190) | 122 (39- 204) | 127 (52- 201) | 154 (134-175) |
| **Primary homelessness: living on the streets, in parks or in deserted buildings; secondary: living in temporary shelters such as refuge, emergency accommodation, or sleeping on friend's couch; tertiary: private boarding room* | | | | | | |

*Population estimates are presented as percentages or means, with 95% confidence intervals. Comparisons may be considered statistically significant when confidence intervals do not overlap .*

| TABLE 2. MARITAL AND PARENTING STATUS | | | | | | | |
| --- | --- | --- | --- | --- | --- | --- | --- |
|  | Schizophrenia | | Schizoaffective disorder | Bipolar disorder  with psychosis | Depressive  psychosis | Delusional and other nonorganic psychoses | Any psychosis |
|  | N=857 | | N=293 | N=319 | N=81 | N=92 | N=1642 |
|  | % (95%CI) | | % (95% CI) | % (95% CI) | % (95% CI) | % (95% CI) | % (95% CI) |
| **Marital status** |  | |  |  |  |  |  |
| Single, never married/de facto |  | |  |  |  |  |  |
| Males | 77.6 (73.8-81.0) | | 63.9 (55.5-71.6) | 56.8 (48.0-65.2) | 64.5 (46.0-79.6) | 79.5 (67.0-88.0) | 72.4 (69.3-75.3) |
| Females | 53.7 (47.1-60.3) | | 39.2 (31.0-48.0) | 34.2 (27.3-41.8) | 34.4 (21.8-49.7) | 56.4 (36.6-74.4) | 44.0 (40.0-48.1) |
| Persons | 70.6 (67.2-73.8) | | 52.5 (46.4-58.5) | 44.3 (38.6-50.0) | 46.4 (35.2-57.9) | 73.0 (62.5-81.5) | 61.3 (58.8-63.8) |
| Currently married, de facto |  | |  |  |  |  |  |
| Males | 8.3 (6.2-11.0) | | 20.4 (14.4-28.0) | 20.4 (14.0-28.7) | 16.3 (7.0-33.4) | 8.5 (3.8-17.9) | 12.0 (10.0-14.4) |
| Females | 19.5 (14.7-25.4) | | 26.6 (19.3-35.3) | 31.5 (24.7-39.2) | 30.0 (18.5-44.9) | 19.6 (8.3-39.5) | 25.1 (21.7-28.9) |
| Persons | 11.6 (9.5-14.2) | | 23.2 (18.4-28.9) | 26.6 (21.7-32.1) | 24.6 (16.2-35.5) | 11.6 (6.5-19.9) | 17.1 (15.3-19.2) |
| **Parental status** |  | |  |  |  |  |  |
| Children of any age (own) |  | |  |  |  |  |  |
| Males | 23.5 (20.0-27.3) | | 30.4 (23.2-38.7) | 32.2 (24.6-41.0) | 47.2 (30.0-65.0) | 22.6 (13.5-35.2) | 26.4 (23.6-29.5) |
| Females | 51.5 (44.9-58.0) | | 60.0 (51.1-68.3) | 61.0 (53.3-68.2) | 63.8 (48.6-76.6) | 66.9 (45.5-83.1) | 57.4 (53.3-61.5) |
| Persons | 31.7 (28.5-35.2) | | 44.1 (38.2-50.3) | 48.2 (42.4-54.0) | 57.2 (45.6-68.0) | 35.0 (25.4-45.9) | 38.5 (36.0-41.0) |
| Children under 18 years living at home (own or step) | |  |  |  |  |  |  |
| Males | 3.5 (2.2-5.5) | | 6.7 (3.4-12.7) | 10.4 (6.1-17.2) | 16.4 (7.0-33.7) | 5.9 (2.1-15.7) | 5.6 (4.2-7.3) |
| Females | 19.7 (15.1-25.4) | | 25.9 (18.8-34.4) | 24.1 (18.1-31.3) | 32.2 (20.1-47.2) | 19.6 (7.8-41.2) | 23.2 (19.9-26.8) |
| Persons | 8.3 (6.6-10.5) | | 15.6 (11.6-20.6) | 18.0 (14.0-22.9) | 25.9 (17.2-37.0) | 9.7 (4.9-18.5) | 12.4 (10.8-14.2) |
|  | | | | | | | |

*Population estimates are presented as percentages or means, with 95% confidence intervals. Comparisons may be considered statistically significant when confidence intervals do not overlap .*

| TABLE 3. MENTAL HEALTH PROFILE | | | | | | |
| --- | --- | --- | --- | --- | --- | --- |
|  | Schizophrenia | Schizoaffective disorder | Bipolar disorder  with psychosis | Depressive  psychosis | Delusional and other nonorganic psychoses | Any psychosis |
|  | N=857 | N=293 | N=319 | N=81 | N=92 | N=1642 |
|  | % (95%CI) | % (95% CI) | % (95% CI) | % (95% CI) | % (95% CI) | % (95% CI) |
| **Onset, duration and course** |  |  |  |  |  |  |
| Onset within two years prior to interview | 8.3 (6.7-10.3) | 4.3 (2.6-7.1) | 7.1 (4.8-10.3) | 7.6 (3.0-17.6) | 10.0 (5.5-17.5) | 7.5 (6.3-8.8) |
| Age at onset (mean years; 95%CI) | 24 (23-24) | 23 (22-24) | 25 (24-26) | 25 (22-28) | 24 (22-25) | 24 (23-24) |
| Duration (mean years; 95%CI) | 15 (14-16) | 16 (15-17) | 17 (16-18) | 16 (13-19) | 16 (13-18) | 16 (15-16) |
| Course of disorder |  |  |  |  |  |  |
| Single episode | 6.4 (5.0-8.2) | 3.2 (1.6-6.3) | 3.5 (2.0-6.0) | 5.3 (1.9-14.3) | 16.5 (9.8-26.6) | 5.9 (4.8-7.1) |
| Multiple episodes  with good/partial recovery between | 54.8 (51.3-58.3) | 65.5 (59.4-71.1) | 77.6 (72.5-82.0) | 63.4 (51.9-73.6) | 57.3 (46.2-67.6) | 61.6 (59.1-64.1) |
| Continuous chronic illness  with/without deterioration | 38.8 (35.4-42.3) | 31.3 (25.9-37.3) | 18.8 (14.8-23.8) | 31.2 (21.8-42.5) | 26.2 (17.7-37.0) | 32.5 (30.1-35.0) |
| TOTAL | 100.0 | 100.0 | 100.0 | 100.0 | 100.0 | 100.0 |
| **Key symptoms (past year)** |  |  |  |  |  |  |
| Delusions | 67.4 (64.0-70.7) | 70.7 (64.7-76.0) | 50.7 (44.9-56.4) | 61.7 (50.1-72.1) | 45.7 (35.2-56.6) | 63.1 (60.6-65.5) |
| Hallucinations | 63.6 (60.1-67.0) | 66.9 (60.9-72.5) | 38.3 (32.9-44.1) | 50.0 (38.6-61.3) | 39.6 (29.7-50.4) | 57.1 (54.5-59.6) |
| Subjective thought disorder (thought broadcast or insertion and "loud thoughts") | 35.1 (31.8-38.6) | 48.0 (42.0-54.0) | 19.3 (15.1-24.3) | 33.6 (23.6-45.3) | 14.3 (8.5-23.1) | 32.8 (30.5-35.2) |
| Elevated or irritable mood | 11.3 (9.3-13.7) | 34.4 (29.0-40.3) | 57.2 (51.4-62.8) | 8.8 (4.3-17.3) | 7.2 (3.3-15.2) | 23.7 (21.6-25.9) |
| Depressed mood or loss of pleasure | 37.5 (34.1-41.0) | 65.7 (59.7-71.3) | 64.0 (58.1-69.4) | 95.7 (88.4-98.5) | 44.8 (34.4-55.6) | 50.8 (48.2-53.3) |
| Symptoms of anxiety/phobia | 54.9 (51.4-58.4) | 63.0 (56.9-68.6) | 64.2 (58.5-69.6) | 77.2 (66.0-85.6) | 45.8 (35.3-56.7) | 58.7 (56.1-61.2) |
| **Negative symptoms (past year)** |  |  |  |  |  |  |
| Dysfunction in overall socialising | 67.0 (63.6-70.2) | 61.6 (55.7-67.1) | 58.1 (52.3-63.8) | 79.4 (68.3-87.3) | 68.0 (57.0-77.3) | 65.1 (62.6-67.5) |
| Diminished sense of purpose | 56.3 (52.7-59.8) | 52.8 (46.7-58.8) | 44.2 (38.5-50.0) | 65.7 (54.0-75.7) | 46.9 (36.3-57.8) | 53.3 (50.7-55.8) |
| Lack of interest and motivation | 53.8 (50.2-57.3) | 52.7 (46.6-58.6) | 51.8 (46.0-57.6) | 66.3 (54.7-76.2) | 52.5 (41.6-63.2) | 53.8 (51.2-56.3) |
| Diminished emotional range | 56.5 (53.0-60.0) | 46.8 (40.8-52.9) | 45.2 (39.5-51.0) | 64.9 (53.3-75.0) | 53.5 (42.6-64.0) | 53.0 (50.4-55.5) |
| Restricted affect | 54.2 (50.6-57.7) | 34.2 (28.6-40.4) | 30.5 (25.4-36.2) | 60.1 (48.5-70.8) | 48.6 (37.9-59.5) | 46.2 (43.7-48.8) |
| Poverty of speech | 26.2 (23.2-29.4) | 14.6 (10.6-19.7) | 7.3 (4.9-10.7) | 9.5 (4.9-17.7) | 20.5 (13.1-30.6) | 19.4 (17.4-21.5) |
| Any of six negative symptoms | 88.4 (86.0-90.5) | 82.7 (78.1-86.6) | 82.2 (77.2-86.2) | 96.3 (90.4-98.6) | 87.0 (77.4-92.9) | 86.6 (84.8-88.2) |
| Four or more negative symptoms | 45.9 (42.4-49.5) | 33.5 (27.9-39.7) | 29.2 (24.2-34.7) | 59.8 (48.2-70.5) | 44.2 (33.7-55.2) | 41.2 (38.7-43.8) |
| **Suicidal ideation** |  |  |  |  |  |  |
| Suicidal ideation (past year) | 20.2 (17.5-23.1) | 34.8 (29.2-40.7) | 33.3 (28.1-39.0) | 71.8 (60.3-81.0) | 18.2 (11.5-27.5) | 27.7 (25.5-30.0) |
| Suicide attempt (ever) | 42.6 (39.1-46.1) | 61.4 (55.4-67.0) | 53.0 (47.2-58.7) | 72.7 (61.3-81.7) | 40.2 (30.2-51.0) | 49.1 (46.6-51.7) |
|  | | | | | | |

*Population estimates are presented as percentages or means, with 95% confidence intervals. Comparisons may be considered statistically significant when confidence intervals do not overlap .*

| TABLE 4. PHYSICAL HEALTH PROFILE | | | | | | |
| --- | --- | --- | --- | --- | --- | --- |
|  | Schizophrenia | Schizoaffective disorder | Bipolar disorder  with psychosis | Depressive psychosis | Delusional and other nonorganic psychoses | Any psychosis |
|  | N=857 | N=293 | N=319 | N=81 | N=92 | N=1642 |
|  | % (95%CI) | % (95% CI) | % (95% CI) | % (95% CI) | % (95% CI) | % (95% CI) |
| **Cardiometabolic risk profile** |  |  |  |  |  |  |
| Metabolic syndrome* | 58.3 (54.2-62.4) | 63.3 (56.5-69.7) | 67.4 (60.9-73.3) | 52.4 (39.9-64.6) | 62.2 (49.5-73.4) | 60.8 (57.9-63.7) |
| *Met threshold criteria for component risks for metabolic syndrome :* | | | | | | |
| Abdominal obesity | 83.1 (80.3-85.5) | 83.3 (78.4-87.3) | 87.6 (83.3-90.9) | 87.9 (78.6-93.5) | 82.1 (72.6-88.8) | 84.2 (82.3-86.0) |
| Reduced high density lipoprotein levels | 58.0 (53.8-62.1) | 59.4 (52.5-66.0) | 58.1 (51.3-64.6) | 48.4 (36.1-60.9) | 64.9 (52.0-76.0) | 58.1 (55.1-61.0) |
| Elevated triglyceride levels | 56.0 (51.8-60.1) | 54.9 (48.0-61.7) | 56.0 (49.2-62.6) | 50.3 (37.8-62.7) | 55.9 (43.1-67.8) | 55.5 (52.5-58.4) |
| Elevated glucose levels | 35.7 (31.7-39.9) | 37.4 (30.8-44.6) | 35.6 (29.3-42.5) | 31.6 (20.9-44.7) | 28.2 (18.2-41.1) | 35.3 (32.4-38.3) |
| Elevated blood pressure | 51.7 (48.1-55.3) | 53.5 (47.4-59.5) | 59.6 (53.7-65.1) | 52.4 (40.6-63.9) | 64.4 (53.3-74.2) | 54.4 (51.8-56.9) |
| **Lifestyle risk factors for cardiovascular disease** |  |  |  |  |  |  |
| Current smoking | 67.0 (63.6-70.3) | 70.3 (64.4-75.6) | 59.3 (53.5-64.9) | 55.8 (44.1-66.8) | 73.8 (63.5-82.1) | 65.9 (63.4-68.3) |
| Body mass index: |  |  |  |  |  |  |
| Underweight | 1.0 (0.5-1.9) | 1.8 (0.8-4.0) | 0.8 (0.3-2.5) | 1.9 (0.5-7.5) | 2.4 (0.8-7.4) | 1.2 (0.8-1.8) |
| Normal | 20.9 (18.2-23.8) | 18.2 (14.0-23.2) | 17.9 (14.0-22.7) | 16.3 (9.5-26.5) | 22.1 (14.3-32.5) | 19.7 (17.8-21.7) |
| Overweight | 29.0 (25.8-32.3) | 29.1 (23.9-34.8) | 27.9 (23.1-33.3) | 25.5 (16.5-37.3) | 35.9 (26.1-47.2) | 29.0 (26.7-31.4) |
| Obese | 46.0 (42.5-49.6) | 49.0 (43.0-55.0) | 51.2 (45.4-57.0) | 52.3 (40.8-63.5) | 38.9 (28.9-49.8) | 47.4 (44.9-50.0) |
| Missing | 3.2 (2.1-4.7) | 2.0 (0.8-4.6) | 2.1 (1.0-4.4) | 4.0 (1.3-12.0) | 0.7 (0.1-4.8) | 2.7 (2.0-3.6) |
| TOTAL | 100.0 | 100.0 | 100.0 | 100.0 | 100.0 | 100.0 |
| Level of physical activity: |  |  |  |  |  |  |
| Very low | 32.2 (29.0-35.6) | 36.2 (30.6-42.2) | 32.3 (27.2-38.0) | 31.6 (22.3-42.8) | 24.1 (16.1-34.3) | 32.4 (30.1-34.8) |
| Low | 63.8 (60.3-67.1) | 58.9 (52.8-64.7) | 63.8 (58.1-69.2) | 66.4 (55.1-76.1) | 72.1 (61.6-80.6) | 63.6 (61.1-66.0) |
| Moderate | 3.3 (2.2-4.9) | 4.2 (2.3-7.3) | 3.4 (1.7-6.6) | 2.0 (0.3-12.7) | 0.7 (0.1-4.8) | 3.3 (2.4-4.3) |
| High | 0.5 (0.1-1.5) | 0.3 (0.0-1.8) | 0.2 (0.0-1.6) | 0.0 | 0.7 (0.1-5.0) | 0.4 (0.2-0.9) |
| Missing | 0.2 (0.0-1.4) | 0.5 (0.1-2.0) | 0.2 (0.0-1.6) | 0.0 | 2.4 (0.5-10.4) | 0.4 (0.2-0.9) |
| TOTAL | 100.0 | 100.0 | 100.0 | 100.0 | 100.0 | 100.0 |
| One or fewer serves of vegetables a day | 47.3 (43.8-50.8) | 48.4 (42.3-54.4) | 47.0 (41.3-52.8) | 52.5 (41.0-63.7) | 54.3 (43.4-64.8) | 48.1 (45.5-50.7) |
| One or fewer serves of fruit a day | 71.2 (67.8-74.3) | 67.4 (61.5-72.8) | 70.1 (64.7-75.0) | 72.3 (60.6-81.5) | 74.4 (64.3-82.4) | 70.6 (68.2-72.9) |
| **Other physical health conditions** |  |  |  |  |  |  |
| Asthma | 28.0 (24.9-31.3) | 34.1 (28.6-40.0) | 32.2 (27.1-37.8) | 43.0 (32.2-54.6) | 18.3 (11.5-27.9) | 30.0 (27.7-32.4) |
| Cardiovascular disease: incl. high blood pressure | 26.7 (23.6-30.0) | 28.7 (23.6-34.4) | 30.1 (25.0-35.7) | 32.1 (22.4-43.6) | 26.3 (17.8-36.9) | 27.9 (25.7-30.3) |
| Cardiovascular disease: excl. high blood pressure | 11.5 (9.3-14.1) | 15.9 (12.1-20.6) | 11.1 (8.0-15.3) | 17.9 (10.5-29.0) | 7.8 (3.5-16.9) | 12.2 (10.6-14.1) |
| Severe headaches/migraines | 20.1 (17.4-23.1) | 29.1 (23.9-34.9) | 32.4 (27.3-38.1) | 39.1 (28.6-50.7) | 17.7 (11.2-26.8) | 24.8 (22.7-27.1) |
| Diabetes | 21.6 (18.8-24.8) | 22.9 (18.2-28.4) | 22.4 (17.8-27.7) | 16.4 (9.8-26.3) | 15.9 (9.6-25.2) | 21.4 (19.3-23.6) |
| Arthritis | 18.9 (16.2-21.9) | 21.4 (16.9-26.7) | 26.0 (21.2-31.4) | 37.1 (26.7-48.9) | 12.3 (6.8-21.3) | 21.2 (19.2-23.5) |
| Respiratory conditions | 17.9 (15.3-20.8) | 20.2 (15.9-25.4) | 16.1 (12.3-20.7) | 33.2 (23.1-45.2) | 8.7 (4.4-16.4) | 18.2 (16.3-20.2) |
| Anaemia | 9.8 (7.9-12.2) | 16.8 (12.6-22.0) | 17.0 (13.1-21.8) | 29.3 (20.0-40.8) | 6.2 (3.0-12.1) | 13.2 (11.5-15.0) |
| Hepatitis | 11.7 (9.6-14.2) | 12.3 (8.6-17.1) | 11.4 (8.1-15.9) | 10.9 (5.5-20.5) | 16.8 (9.9-27.1) | 12.0 (10.4-13.8) |
| Epilepsy | 7.9 (6.2-10.1) | 7.4 (4.8-11.3) | 6.1 (4.0-9.3) | 7.8 (3.4-17.2) | 7.0 (3.3-14.3) | 7.4 (6.2-8.9) |
| * *Percentages are for participants with valid fasting blood measures.* | | | | | | |

*Population estimates are presented as percentages or means, with 95% confidence intervals. Comparisons may be considered statistically significant when confidence intervals do not overlap .*

| TABLE 5. COGNITIVE PROFILE | | | | | | |
| --- | --- | --- | --- | --- | --- | --- |
|  | Schizophrenia | Schizoaffective disorder | Bipolar disorder  with psychosis | Depressive  psychosis | Delusional and other nonorganic psychoses | Any psychosis |
|  | Mean, SD  (95%CI) | Mean, SD  (95%CI) | Mean, SD  (95%CI) | Mean, SD  (95%CI) | Mean, SD  (95%CI) | Mean, SD  (95%CI) |
| **Premorbid IQ* - NART-R** | | | | | | |
|  | N=704 | N=250 | N=292 | N=73 | N=72 | N=1391 |
|  | 96.6, 11.3 (95.4-97.8) | 99.6, 11.0 (97.3-102.0) | 100.5, 10.8 (98.5-102.6) | 99.2, 12.0 (95.4-103.0) | 95.3, 11.3 (91.7-99.0) | 98.0, 11.3 (96.7-99.3) |
| **Speed of information processing (current)* - Digit Symbol Coding task** | | | | | | |
|  | N=748 | N=261 | N=299 | N=70 | N=78 | N=1456 |
|  | 37.3, 10.2 (35.9-38.7) | 37.6, 10.2 (35.4-39.7) | 40.8, 11.1 (38.8-42.7) | 42.1, 11.4 (39.4-44.8) | 37.1, 9.9 (34.2-39.9) | 38.3, 10.6 (36.9-39.7) |
| *^*^ Data are for 1391 participants with valid NART-R results and 1456 participants with valid Digit Symbol Coding task results.* | | | | | | |

*Population estimates are presented as percentages or means, with 95% confidence intervals. Comparisons may be considered statistically significant when confidence intervals do not overlap .*

| TABLE 6. SMOKING | | | | | | |
| --- | --- | --- | --- | --- | --- | --- |
|  | Schizophrenia | Schizoaffective disorder | Bipolar disorder  with psychosis | Depressive  psychosis | Delusional and other nonorganic psychoses | Any psychosis |
|  | N=857 | N=293 | N=319 | N=81 | N=92 | N=1642 |
|  | % (95%CI) | % (95% CI) | % (95% CI) | % (95% CI) | % (95% CI) | % (95% CI) |
| **Current smoking** |  |  |  |  |  |  |
| Males | 72.3 (68.3-76.0) | 72.0 (63.8-78.9) | 59.8 (51.1-68.0) | 63.3 (44.1-79.1) | 73.7 (61.1-83.3) | 70.3 (67.2-73.2) |
| Females | 54.5 (47.9-61.0) | 68.3 (59.5-75.9) | 58.9 (51.0-66.3) | 50.8 (36.3-65.2) | 74.3 (54.6-87.4) | 59.0 (54.8-63.0) |
| Persons | 67.0 (63.6-70.3) | 70.3 (64.4-75.6) | 59.3 (53.5-64.9) | 55.8 (44.1-66.8) | 73.8 (63.5-82.1) | 65.9 (63.4-68.3) |
| **Fagerstrom Nicotine Dependence Scale (current)** | | | | | |  |
| Males: |  |  |  |  |  |  |
| Very low | 32.3 (28.5-36.4) | 33.0 (25.6-41.3) | 44.0 (35.6-52.7) | 57.8 (40.0-73.7) | 37.7 (26.1-50.9) | 35.3 (32.2-38.5) |
| Low | 12.5 (10.0-15.5) | 9.4 (5.8-15.0) | 10.7 (6.5-17.2) | 4.5 (0.6-25.7) | 13.3 (6.5-25.6) | 11.6 (9.7-13.8) |
| Moderate | 10.3 (8.1-13.1) | 11.6 (7.2-18.2) | 11.8 (7.3-18.7) | 6.2 (1.9-17.8) | 9.6 (4.4-19.8) | 10.5 (8.7-12.7) |
| High | 24.9 (21.4-28.6) | 20.1 (14.3-27.4) | 15.4 (10.1-22.8) | 20.2 (9.8-37.0) | 23.6 (14.3-36.3) | 22.6 (20.0-25.4) |
| Very high | 20.0 (16.9-23.5) | 25.9 (19.4-33.7) | 18.0 (12.0-26.1) | 11.4 (4.6-25.4) | 15.8 (8.7-27.2) | 20.0 (17.5-22.7) |
| TOTAL | 100.0 | 100.0 | 100.0 | 100.0 | 100.0 | 100.0 |
| Females: |  |  |  |  |  |  |
| Very low | 53.5 (46.9-60.0) | 35.5 (27.5-44.4) | 48.0 (40.4-55.8) | 61.4 (46.2-74.7) | 34.6 (18.6-55.0) | 48.3 (44.2-52.4) |
| Low | 8.1 (5.2-12.4) | 6.8 (3.3-13.5) | 10.4 (6.7-15.9) | 3.3 (0.5-19.8) | 10.9 (4.0-26.4) | 8.2 (6.2-10.7) |
| Moderate | 5.5 (3.3-9.2) | 10.9 (6.5-17.7) | 7.6 (4.4-12.8) | 1.8 (0.3-12.0) | 12.4 (3.8-33.9) | 7.1 (5.3-9.5) |
| High | 18.1 (13.6-23.8) | 27.2 (20.1-35.6) | 22.5 (16.7-29.5) | 19.0 (9.9-33.4) | 15.8 (5.1-39.5) | 21.1 (17.9-24.6) |
| Very high | 14.8 (10.7-20.0) | 19.7 (13.4-27.9) | 11.5 (7.4-17.3) | 14.5 (6.8-28.4) | 26.4 (12.4-47.5) | 15.3 (12.6-18.5) |
| TOTAL | 100.0 | 100.0 | 100.0 | 100.0 | 100.0 | 100.0 |
| Persons: |  |  |  |  |  |  |
| Very low | 38.6 (35.2-42.1) | 34.2 (28.6-40.2) | 46.2 (40.5-52.1) | 60.0 (48.5-70.5) | 36.8 (27.0-47.8) | 40.4 (37.9-42.9) |
| Low | 11.2 (9.2-13.6) | 8.2 (5.5-12.1) | 10.6 (7.6-14.5) | 3.8 (0.9-13.8) | 12.7 (7.0-21.9) | 10.3 (8.8-11.9) |
| Moderate | 8.9 (7.1-11.1) | 11.3 (7.9-15.8) | 9.5 (6.6-13.4) | 3.6 (1.3-9.3) | 10.4 (5.4-19.0) | 9.2 (7.9-10.8) |
| High | 22.9 (20.1-26.0) | 23.4 (18.7-28.8) | 19.3 (15.2-24.3) | 19.5 (12.1-29.8) | 21.4 (13.6-32.0) | 22.0 (20.0-24.2) |
| Very high | 18.4 (15.9-21.3) | 23.0 (18.3-28.5) | 14.4 (10.7-19.1) | 13.3 (7.4-22.6) | 18.8 (11.8-28.6) | 18.1 (16.3-20.2) |
| TOTAL | 100.0 | 100.0 | 100.0 | 100.0 | 100.0 | 100.0 |
|  | | | | | | |

*Population estimates are presented as percentages or means, with 95% confidence intervals. Comparisons may be considered statistically significant when confidence intervals do not overlap .*

| TABLE 7. ALCOHOL USE | | | | | | |
| --- | --- | --- | --- | --- | --- | --- |
|  | Schizophrenia | Schizoaffective disorder | Bipolar disorder  with psychosis | Depressive  psychosis | Delusional and other nonorganic psychoses | Any psychosis |
|  | N=857 | N=293 | N=319 | N=81 | N=92 | N=1642 |
|  | % (95%CI) | % (95% CI) | % (95% CI) | % (95% CI) | % (95% CI) | % (95% CI) |
| **Lifetime alcohol abuse/dependence** |  |  |  |  |  |  |
| Males | 58.1 (53.9-62.2) | 51.1 (42.8-59.2) | 57.7 (48.9-65.9) | 59.0 (40.3-75.4) | 60.3 (47.2-72.1) | 57.2 (53.9-60.4) |
| Females | 33.2 (27.3-39.6) | 41.3 (32.8-50.4) | 40.6 (33.2-48.3) | 45.6 (31.5-60.4) | 38.0 (20.6-59.1) | 38.0 (34.1-42.1) |
| Persons | 50.7 (47.2-54.3) | 46.5 (40.5-52.6) | 48.2 (42.4-54.0) | 50.9 (39.5-62.2) | 54.1 (43.2-64.6) | 49.8 (47.2-52.3) |
| **Alcohol Use Disorders Identification Test (past year)** | | | | | | |
| Males |  |  |  |  |  |  |
| Low risk | 67.3 (63.3-71.0) | 65.7 (57.4-73.1) | 60.1 (51.3-68.3) | 56.5 (38.3-73.0) | 68.0 (54.9-78.7) | 65.7 (62.6-68.7) |
| Hazardous drinking | 19.4 (16.4-22.8) | 15.0 (10.0-21.9) | 18.4 (12.6-26.0) | 11.0 (3.8-28.2) | 15.3 (8.2-26.6) | 18.0 (15.7-20.6) |
| Harmful drinking | 5.4 (3.9-7.5) | 7.4 (4.0-13.2) | 11.7 (7.0-19.0) | 2.0 (0.3-13.0) | 5.2 (1.7-15.0) | 6.5 (5.1-8.2) |
| Dependent drinking | 7.9 (5.9-10.5) | 11.9 (7.6-18.3) | 9.8 (5.6-16.5) | 30.5 (16.6-49.3) | 11.6 (5.4-23.1) | 9.8 (8.0-11.9) |
| TOTAL | 100.0 | 100.0 | 100.0 | 100.0 | 100.0 | 100.0 |
| Females |  |  |  |  |  |  |
| Low risk | 82.2 (76.8-86.5) | 71.1 (62.3-78.5) | 76.6 (69.4-82.5) | 72.4 (57.2-83.8) | 67.6 (46.0-83.6) | 77.0 (73.4-80.3) |
| Hazardous drinking | 11.4 (8.0-16.0) | 20.6 (14.1-29.0) | 17.1 (12.0-23.8) | 12.7 (5.8-25.4) | 20.5 (8.2-42.7) | 15.3 (12.6-18.4) |
| Harmful drinking | 1.4 (0.5-3.9) | 5.2 (2.4-10.8) | 0.9 (0.2-3.5) | 4.3 (1.0-16.9) | 8.7 (1.9-31.3) | 2.5 (1.5-4.2) |
| Dependent drinking | 5.1 (2.9-8.8) | 3.2 (1.3-7.5) | 5.4 (2.9-10.0) | 10.6 (4.0-25.4) | 3.2 (0.4-19.9) | 5.2 (3.6-7.3) |
| TOTAL | 100.0 | 100.0 | 100.0 | 100.0 | 100.0 | 100.0 |
| Persons |  |  |  |  |  |  |
| Low risk | 71.7 (68.5-74.7) | 68.2 (62.3-73.6) | 69.2 (63.6-74.4) | 66.1 (54.5-76.1) | 67.9 (56.9-77.2) | 70.1 (67.8-72.4) |
| Hazardous drinking | 17.0 (14.6-19.7) | 17.6 (13.4-22.8) | 17.7 (13.7-22.5) | 12.0 (6.4-21.4) | 16.7 (10.1-26.5) | 17.0 (15.2-18.9) |
| Harmful drinking | 4.2 (3.1-5.8) | 6.4 (4.0-10.1) | 5.7 (3.5-9.2) | 3.4 (1.0-10.8) | 6.1 (2.5-14.4) | 4.9 (4.0-6.1) |
| Dependent drinking | 7.1 (5.5-9.1) | 7.9 (5.2-11.7) | 7.3 (4.8-11.0 ) | 18.5 (11.0-29.5) | 9.2 (4.5-18.0) | 8.0 (6.7-9.5) |
| TOTAL | 100.0 | 100.0 | 100.0 | 100.0 | 100.0 | 100.0 |
|  |  |  |  |  |  |  |

*Population estimates are presented as percentages or means, with 95% confidence intervals. Comparisons may be considered statistically significant when confidence intervals do not overlap .*

| TABLE 8. SUBSTANCE USE | | | | | | |
| --- | --- | --- | --- | --- | --- | --- |
|  | Schizophrenia | Schizoaffective disorder | Bipolar disorder  with psychosis | Depressive  psychosis | Delusional and other nonorganic psychoses | Any psychosis |
|  | N=857 | N=293 | N=319 | N=81 | N=92 | N=1642 |
|  | % (95%CI) | % (95% CI) | % (95% CI) | % (95% CI) | % (95% CI) | % (95% CI) |
| **Cannabis abuse/dependence** |  |  |  |  |  |  |
| Lifetime cannabis abuse/dependence |  |  |  |  |  |  |
| Males | 62.4 (58.2-66.4) | 58.3 (50.0-66.2) | 59.1 (50.4-67.3) | 47.7 (30.6-65.5) | 52.8 (39.9-65.3) | 60.2 (56.9-63.4) |
| Females | 34.3 (28.3-40.8) | 37.4 (29.2-46.4) | 38.8 (31.6-46.5) | 35.8 (22.8-51.2) | 31.2 (15.3-53.4) | 36.2 (32.3-40.2) |
| Persons | 54.1 (50.5-57.6) | 48.6 (42.6-54.7) | 47.8 (42.1-53.6) | 40.5 (29.9-52.2) | 46.8 (36.2-57.7) | 50.8 (48.3-53.4) |
| Any cannabis use (past year) |  |  |  |  |  |  |
| Males | 35.7 (31.9-39.8) | 41.8 (33.9-50.1) | 33.2 (25.7-41.7) | 46.9 (29.9-64.7) | 35.6 (24.3-48.8) | 36.6 (33.6-39.8) |
| Females | 16.2 (12.2-21.3) | 26.3 (19.2-34.8) | 26.2 (20.2-33.2) | 23.5 (13.1-38.5) | 17.6 (6.3-40.3) | 21.6 (18.5-25.1) |
| Persons | 30.0 (26.9-33.2) | 34.6 (29.1-40.5) | 29.3 (24.5-34.7) | 32.8 (23.0-44.3) | 30.6 (21.4-41.6) | 30.8 (28.5-33.1) |
| Almost daily cannabis use (past year) |  |  |  |  |  |  |
| Males | 12.3 (9.9-15.2) | 19.8 (14.0-27.3) | 17.4 (11.9-24.8) | 20.8 (9.7-39.2) | 13.5 (6.8-24.9) | 14.5 (12.4-16.9) |
| Females | 4.3 (2.4-7.8) | 10.8 (6.4-17.8) | 10.5 (7.0-15.5) | 8.7 (3.1-21.8) | 8.8 (2.0-31.8) | 7.9 (6.0-10.3) |
| Persons | 10.0 (8.1-12.2) | 15.6 (11.7-20.6) | 13.6 (10.3-17.8) | 13.5 (7.3-23.5) | 12.2 (6.6-21.4) | 11.9 (10.4-13.6) |
| **Other drug abuse/dependence** |  |  |  |  |  |  |
| Lifetime other drug abuse/dependence |  |  |  |  |  |  |
| Males | 36.3 (32.4-40.4) | 39.0 (31.3-47.3) | 34.3 (26.6-42.9) | 24.7 (13.1-41.8) | 36.1 (24.6-49.4) | 36.0 (33.0-39.2) |
| Females | 21.0 (16.3-26.7) | 28.4 (21.1-36.9) | 24.6 (18.7-31.7) | 18.3 (9.4-32.8) | 23.2 (9.7-45.8) | 23.4 (20.1-26.9) |
| Persons | 31.8 (28.7-35.1) | 34.1 (28.6-40.0) | 28.9 (24.0-34.4) | 20.9 (13.2-31.3) | 32.5 (23.0-43.7) | 31.1 (28.8-33.5) |
| **Amphetamine use** |  |  |  |  |  |  |
| Amphetamine use, past year | 12.1 (10.0-14.5) | 13.7 (10.0-18.4) | 13.5 (10.1-17.9) | 6.1 (2.9-12.6) | 8.4 (4.2-16.1) | 12.1 (10.6-13.8) |
| Amphetamine use, lifetime | 39.1 (35.7-42.6) | 44.9 (39.0-51.0) | 38.2 (32.8-44.0) | 30.2 (20.8-41.6) | 43.3 (32.9-54.5) | 39.7 (37.2-42.2) |
|  |  |  |  |  |  |  |

*Population estimates are presented as percentages or means, with 95% confidence intervals. Comparisons may be considered statistically significant when confidence intervals do not overlap .*

| TABLE 9. FUNCTIONING, QUALITY OF LIFE AND SOCIAL RELATIONSHIPS | | | | | | |
| --- | --- | --- | --- | --- | --- | --- |
|  | Schizophrenia | Schizoaffective disorder | Bipolar disorder  with psychosis | Depressive psychosis | Delusional and other nonorganic psychoses | Any psychosis |
|  | N=857 | N=293 | N=319 | N=81 | N=92 | N=1642 |
|  | % (95%CI) | % (95% CI) | % (95% CI) | % (95% CI) | % (95% CI) | % (95% CI) |
| **Functioning** |  |  |  |  |  |  |
| Good premorbid work adjustment | 67.2 (63.8-70.4) | 72.2 (66.4-77.3) | 72.9 (67.5-77.8) | 68.0 (56.7-77.6) | 64.8 (54.0-74.2) | 69.0 (66.6-71.3) |
| Good premorbid social adjustment | 61.3 (57.8-64.7) | 67.7 (61.7-73.1) | 67.3 (61.7-72.5) | 57.4 (45.8-68.3) | 64.6 (53.8-74.1) | 63.5 (61.0-66.0) |
| Deterioration from premorbid level of functioning | 93.2 (91.2-94.8) | 95.6 (92.4-97.5) | 86.5 (81.9-90.1) | 88.6 (78.8-94.2) | 89.4 (80.1-94.6) | 91.8 (90.3-93.2) |
| Obvious/severe dysfunction in social drive, past year | 67.0 (63.6-70.2) | 61.6 (55.7-67.1) | 58.1 (52.3-63.8) | 79.4 (68.3-87.3) | 68.0 (57.0-77.3) | 65.1 (62.6-67.5) |
| Obvious/severe dysfunction in self care, past 4 weeks | 37.8 (34.4-41.3) | 28.5 (23.2-34.4) | 28.8 (23.8-34.3) | 39.8 (29.2-51.5) | 32.3 (22.9-43.4) | 34.3 (31.9-36.8) |
| (Almost) daily face-to-face family contact, past year | 51.5 (47.9-55.0) | 61.7 (55.7-67.4) | 62.3 (56.5-67.8) | 56.3 (44.7-67.2) | 45.9 (35.4-56.8) | 55.2 (52.6-57.7) |
| Has no friends currently | 15.7 (13.3-18.5) | 10.8 (7.6-15.1) | 11.7 (8.4-16.1) | 19.8 (12.2-30.6) | 15.4 (8.8-25.5) | 14.3 (12.6-16.2) |
| Has never had a confiding relationship | 16.5 (14.0-19.3) | 12.6 (9.2-17.1) | 13.8 (10.2-18.3) | 22.1 (14.0-33.1) | 17.1 (10.5-26.7) | 15.7 (13.9-17.6) |
| Global independent performance , past 4 weeks | | | | | | |
| No or very mild disability | 18.2 (15.6-21.1) | 27.3 (22.3-32.9) | 35.1 (29.8-40.8) | 29.2 (19.8-40.8) | 17.6 (11.0-27.1) | 23.5 (21.4-25.8) |
| Somewhat disabled | 26.5 (23.5-29.8) | 22.7 (18.2-28.0) | 23.1 (18.6-28.3) | 35.1 (25.0-46.8) | 27.6 (18.8-38.6) | 25.8 (23.6-28.1) |
| Moderately disabled | 29.6 (26.5-33.0) | 30.9 (25.5-37.0) | 23.6 (19.0-28.9) | 24.5 (15.8-36.0) | 31.8 (22.8-42.5) | 28.5 (26.2-30.9) |
| Significantly, extremely or totally disabled | 25.6 (22.7-28.8) | 19.0 (14.8-24.2) | 18.2 (14.1-23.1) | 11.1 (6.3-18.9) | 22.9 (14.7-33.7) | 22.2 (20.2-24.3) |
| TOTAL | 100.0 | 100.0 | 100.0 | 100.0 | 100.0 | 100.0 |
| Global independent performance* (mean score, 95% CI) | 3.7 (3.5-3.9) | 3.4 (3.1-3.7) | 3.2 (2.9-3.5) | 2.9 (2.9-3.5) | 3.2 (3.3-3.8) | 3.5 (3.4-3.7) |
| **Quality of life** |  |  |  |  |  |  |
| Satisfied with own independence | 75.7 (72.6-78.6) | 64.9 (58.9-70.5) | 71.3 (65.8-76.2) | 59.4 (47.8-70.0) | 78.5 (68.9-85.7) | 72.4 (70.1-74.6) |
| Satisfied with life as a whole | 52.3 (48.7-55.8) | 46.3 (40.3-52.4) | 49.8 (44.1-55.6) | 23.0 (14.6-34.3) | 50.6 (39.8-61.4) | 49.2 (46.7-51.8) |
| * *The higher the score, the greater the disability.* | | | | | | |

*Population estimates are presented as percentages or means, with 95% confidence intervals. Comparisons may be considered statistically significant when confidence intervals do not overlap .*

| TABLE 10. VICTIMISATION AND OFFENDING | | | | | | |
| --- | --- | --- | --- | --- | --- | --- |
|  | Schizophrenia | Schizoaffective disorder | Bipolar disorder  with psychosis | Depressive  psychosis | Delusional and other nonorganic psychoses | Any psychosis |
|  | N=857 | N=293 | N=319 | N=81 | N=92 | N=1642 |
|  | % (95%CI) | % (95% CI) | % (95% CI) | % (95% CI) | % (95% CI) | % (95% CI) |
| **Victimisation, past year** |  |  |  |  |  |  |
| Any victimisation | 35.8 (32.5-39.2) | 40.7 (34.9-46.8) | 39.4 (33.8-45.2) | 46.4 (35.3-57.9) | 42.2 (31.9-53.1) | 38.2 (35.8-40.7) |
| Victim of violence (excluding threatened violence) | 13.1 (11.0-15.6) | 17.9 (13.8-23.0) | 18.9 (14.8-23.8) | 21.0 (13.2-31.8) | 20.3 (12.9-30.6) | 15.9 (14.1-17.8) |
| **Criminal offending, past year** |  |  |  |  |  |  |
| Arrested or charged with an offence | 11.2 (9.2-13.5) | 9.9 (6.9-14.0) | 10.5 (7.4-14.6) | 8.8 (4.1-17.8) | 14.5 (8.6-23.4) | 10.9 (9.5-12.6) |
|  | | | | | | |

*Population estimates are presented as percentages or means, with 95% confidence intervals. Comparisons may be considered statistically significant when confidence intervals do not overlap .*

| TABLE 11. MEDICATION AND MEDICATION SIDE EFFECTS | | | | | | |
| --- | --- | --- | --- | --- | --- | --- |
|  | Schizophrenia | Schizoaffective disorder | Bipolar disorder  with psychosis | Depressive psychosis | Delusional and other nonorganic psychoses | Any psychosis |
|  | N=857 | N=293 | N=319 | N=81 | N=92 | N=1642 |
|  | % (95%CI) | % (95% CI) | % (95% CI) | % (95% CI) | % (95% CI) | % (95% CI) |
| **Medications for mental health, past 4 weeks** |  |  |  |  |  |  |
| Antipsychotics: any | 91.4 (89.1-93.3) | 88.5 (83.9-91.9) | 73.8 (68.4-78.6) | 54.5 (42.9-65.6) | 77.0 (66.7-84.8) | 84.7 (82.7-86.5) |
| Atypical antipsychotics: any | 82.8 (79.9-85.4) | 77.4 (71.9-82.1) | 67.6 (61.9-72.8) | 51.1 (39.7-62.4) | 72.8 (62.2-81.3) | 76.7 (74.4-78.8) |
| Atypical antipsychotics: Clozapine | 26.2 (23.1-29.4) | 14.4 (10.6-19.3) | 5.8 (3.5-9.4) | 2.8 (0.9-8.5) | 15.0 (8.7-24.4) | 18.4 (16.4-20.5) |
| Typical antipsychotics | 18.3 (15.7-21.3) | 19.4 (15.1-24.6) | 11.3 (8.1-15.4) | 7.0 (2.8-16.4) | 11.9 (6.3-21.4) | 16.2 (14.4-18.1) |
| Antidepressants | 29.7 (26.6-33.1) | 40.2 (34.3-46.3) | 40.0 (34.5-45.8) | 68.4 (56.7-78.1) | 42.1 (31.9-53.1) | 36.2 (33.7-38.7) |
| Mood stabilisers | 18.0 (15.3-21.0) | 32.3 (26.9-38.1) | 59.7 (53.9-65.2) | 24.6 (16.0-35.8) | 25.5 (17.2-36.1) | 29.3 (27.0-31.7) |
| Anxiolytics and hypnotics | 14.1 (11.8-16.6) | 18.1 (14.0-23.1) | 20.4 (16.3-25.4) | 18.0 (10.9-28.2) | 13.1 (7.6-21.5) | 16.1 (14.4-18.0) |
| Any medication for mental health | 95.2 (93.4-96.5) | 92.6 (88.5-95.3) | 92.9 (89.3-95.4) | 81.9 (70.9-89.4) | 88.9 (79.3-94.4) | 93.3 (91.8-94.5) |
| Classes of medication used, if any, past 4 weeks* |  |  |  |  |  |  |
| One classes | 45.2 (41.6-48.9) | 27.7 (22.4-33.6) | 21.5 (17.0-26.7) | 29.5 (19.2-42.5) | 34.6 (24.7-46.0) | 36.4 (33.8-38.9) |
| Two classes | 37.3 (33.9-40.9) | 45.9 (39.7-52.2) | 48.2 (42.2-54.2) | 40.7 (29.3-53.3) | 46.1 (35.0-57.5) | 41.5 (38.9-44.1) |
| Three classes | 14.5 (12.0-17.3) | 19.9 (15.4-25.3) | 24.9 (20.1-30.4) | 20.5 (12.3-32.1) | 17.0 (10.1-27.3) | 17.8 (15.9-19.9) |
| Four or more classes | 3.0 (2.0-4.5) | 6.6 (4.1-10.5) | 5.5 (3.5-8.5) | 9.3 (4.0-20.1) | 2.3 (0.5-9.4) | 4.3 (3.4-5.5) |
| TOTAL | 100.0 | 100.0 | 100.0 | 100.0 | 100.0 | 100.0 |
| **Medication side effects, past 4 weeks** |  |  |  |  |  |  |
| Any medication side effects | 79.8 (76.8-82.4) | 83.3 (78.4-87.3) | 79.7 (74.7-84.0) | 72.5 (61.0-81.7) | 74.4 (64.0-82.7) | 79.6 (77.5-81.6) |
| Any impairment due to medication side effects | 62.5 (59.1-65.9) | 69.3 (63.4-74.6) | 64.6 (58.9-70.0) | 63.4 (51.8-73.6) | 45.4 (34.9-56.4) | 63.1 (60.6-65.5) |
| Moderate/severe impairment due to side effects | 30.9 (27.7-34.2) | 33.6 (28.1-39.6) | 32.5 (27.3-38.1) | 32.4 (22.7-43.9) | 20.4 (13.0-30.4) | 31.1 (28.8-33.5) |
| **Percentage of those using psychotropic medications* | | | | | | |

*Population estimates are presented as percentages or means, with 95% confidence intervals. Comparisons may be considered statistically significant when confidence intervals do not overlap .*

| TABLE 12. SERVICE UTILISATION | | | | | | | |
| --- | --- | --- | --- | --- | --- | --- | --- |
|  | Schizophrenia | Schizoaffective disorder | Bipolar disorder  with psychosis | Depressive psychosis | Delusional and other nonorganic psychoses | Any psychosis | |
|  | N=857 | N=293 | N=319 | N=81 | N=92 | N=1642 | |
|  | % (95%CI) | % (95% CI) | % (95% CI) | % (95% CI) | % (95% CI) | % (95% CI) | |
| **Inpatient service utilisation, past year** |  |  |  |  |  |  | |
| Inpatient admission: any | 38.6 (35.2-42.0) | 50.5 (44.5-56.6) | 45.0 (39.3-50.8) | 58.1 (46.5-68.8) | 29.2 (20.4-40.0) | 42.2 (39.7-44.8) | |
| Inpatient admission: mental health | 31.7 (28.6-35.0) | 41.0 (35.2-47.1) | 35.9 (30.6-41.7) | 43.1 (32.1-54.8) | 21.0 (13.5-31.3) | 34.0 (31.6-36.5) | |
| Inpatient admission: physical health | 10.4 (8.4-12.8) | 15.0 (11.2-19.8) | 14.3 (10.7-18.7) | 28.4 (19.1-40.0) | 8.2 (4.0-16.2) | 12.7 (11.1-14.5) | |
| No. of psychiatric admissions, if any (past year): |  |  |  |  |  |  | |
| One admission | 69.3 (63.3-74.6) | 58.5 (49.0-67.5) | 67.3 (57.5-75.8) | 60.5 (41.8-76.5) | 57.4 (34.1-77.8) | 65.7 (61.5-69.7) | |
| Two admissions | 22.7 (17.9-28.3) | 28.2 (20.3-37.7) | 10.8 (6.2-17.9) | 18.9 (8.3-37.3) | 24.6 (10.5-47.6) | 21.1 (17.8-24.9) | |
| Three or more admissions | 8.0 (5.4-11.9) | 13.3 (8.3-20.5) | 22.0 (14.7-31.4) | 20.6 (9.5-39.3) | 18.0 (5.6-45.1) | 13.1 (10.5-16.4) | |
| TOTAL | 100.0 | 100.0 | 100.0 | 100.0 | 100.0 | 100.0 | |
| Total duration, psychiatric admissions (past year): |  |  |  |  |  |  | |
| Less than 2 weeks | 26.0 (20.9-31.8) | 22.9 (16.0-31.6) | 24.3 (16.8-33.6) | 41.9 (25.2-60.6) | 9.6 (2.2-33.6) | 25.4 (21.8-29.4) | |
| 2 weeks - less than 4 weeks | 22.3 (17.6-27.9) | 17.5 (11.7-25.4) | 26.8 (19.0-36.2) | 12.0 (4.6-27.6) | 21.1 (8.3-44.3) | 21.6 (18.2-25.4) | |
| 4 weeks - less than 13 weeks | 40.7 (34.8-46.8) | 50.4 (41.0-59.8) | 42.3 (33.1-52.1) | 33.4 (18.8-52.1) | 53.9 (31.2-75.2) | 43.0 (38.7-47.3) | |
| 13 weeks - less than 26 weeks | 7.0 (4.5-10.7) | 8.4 (4.2-15.8) | 2.7 (0.8-9.1) | 8.9 (2.6-26.6) | 15.4 (4.5-41.2) | 6.8 (4.9-9.4) | |
| 26 - 52 weeks | 4.1 (2.3-7.3) | 0.8 (0.1-5.5) | 3.9 (1.2-11.7) | 3.8 (0.5-22.8) | 0.0 | 3.2 (2.0-5.3) | |
| TOTAL | 100.0 | 100.0 | 100.0 | 100.0 | 100.0 |  | |
| **Involuntary admission, community treatment order, past year** | | | | | | | |
| Involuntary admission | 19.1 (16.6-22.0) | 23.3 (18.7-28.7) | 23.3 (18.7-28.5) | 24.6 (15.7-36.5) | 14.6 (8.5-24.0) | 20.6 (18.7-22.8) | |
| Community treatment order | 25.1 (22.2-28.3) | 19.3 (14.9-24.6) | 16.8 (12.9-21.6) | 4.4 (1.3-13.3) | 19.7 (12.3-29.9) | 21.1 (19.1-23.3) | |
| **Other service utilisation, past year** |  |  |  |  |  |  | |
| Emergency department attendance | 35.2 (31.9-38.6) | 42.8 (37.0-48.8) | 39.8 (34.4-45.6) | 54.3 (42.8-65.4) | 31.0 (22.2-41.6) | 38.1 (35.6-40.6) | |
| Outpatient/community clinic contact: any | 85.0 (82.1-87.5) | 90.1 (85.4-93.4) | 88.1 (83.7-91.4) | 79.4 (68.6-87.3) | 76.9 (65.9-85.2) | 85.7 (83.7-87.5) | |
| Outpatient/community clinic contact: mental health | 81.9 (78.8-84.7) | 86.0 (80.8-90.0) | 83.4 (78.5-87.3) | 71.5 (60.0-80.8) | 71.3 (60.1-80.4) | 81.7 (79.5-83.7) | |
| Outpatient/community clinic contact: physical health | 19.6 (16.9-22.6) | 28.2 (23.0-34.0) | 27.4 (22.5-32.9) | 30.1 (20.5-41.9) | 17.7 (11.0-27.4) | 23.0 (20.9-25.2) | |
| Home visit by mental health team: any | 47.3 (43.8-50.9) | 49.3 (43.3-55.3) | 39.5 (34.0-45.2) | 32.5 (22.8-43.9) | 34.9 (25.5-45.7) | 44.6 (42.1-47.2) | |
| Home visit by mental health team: crisis related | 14.2 (12.0-16.9) | 16.2 (12.1-21.2) | 16.7 (12.9-21.3) | 21.5 (13.6-32.4) | 9.4 (4.7-17.9) | 15.1 (13.4-17.0) | |
| Home visit by mental health team: routine visit | 42.0 (38.6-45.5) | 46.2 (40.3-52.3) | 34.9 (29.6-40.6) | 21.5 (13.6-32.3) | 32.4 (23.3-43.1) | 39.7 (37.2-42.2) | |
| Non-government organisation for mental health | 32.8 (29.5-36.2) | 26.6 (21.5-32.4) | 23.2 (18.6-28.6) | 24.5 (16.2-35.1) | 34.9 (25.2-46.0) | 29.6 (27.3-32.0) | |
| Drug/alcohol services and programs | 11.8 (9.6-14.3) | 11.5 (8.2-15.9) | 12.7 (9.3-17.1) | 22.0 (13.7-33.2) | 18.4 (11.1-28.9) | 12.8 (11.2-14.7) | |
| Case manager: any | 76.8 (73.7-79.7) | 69.4 (63.6-74.6) | 63.6 (57.9-69.0) | 52.4 (40.9-63.6) | 77.1 (67.7-84.3) | 71.8 (69.4-74.0) | |
| Case manager: public mental health case manager | 69.5 (66.1-72.7) | 64.2 (58.3-69.7) | 57.4 (51.6-63.0) | 46.2 (35.0-57.7) | 62.2 (51.3-72.0) | 64.6 (62.1-67.0) | |
| Case manager: NGO case manager | 21.1 (18.4-24.1) | 19.5 (15.1-24.9) | 14.3 (10.6-19.0) | 15.8 (9.4-25.3) | 23.8 (15.6-34.7) | 19.4 (17.4-21.5) | |
| Rehabilitation program: any | 38.6 (35.2-42.1) | 35.6 (30.1-41.6) | 28.9 (24.0-34.3) | 32.4 (22.8-43.8) | 38.5 (28.5-49.6) | 35.9 (33.5-38.4) | |
| Rehabilitation: public mental health program | 13.1 (10.9-15.7) | 15.0 (11.2-19.8) | 13.1 (9.9-17.2) | 14.3 (8.0-24.1) | 11.4 (6.3-19.7) | 13.4 (11.7-15.2) | |
| Rehabilitation : NGO program | 26.5 (23.5-29.7) | 20.2 (15.7-25.6) | 15.6 (11.8-20.3) | 16.8 (9.9-27.0) | 26.3 (17.7-37.2) | 22.8 (20.7-25.1) | |
| **Support and unmet need, past year** |  |  |  |  |  | |  |
| Needed services but unaffordable/unavailable | 23.3 (20.4-26.4) | 27.9 (22.8-33.6) | 35.8 (30.4-41.5) | 52.7 (41.3-63.9) | 24.3 (15.8-35.3) | | 28.1 (25.8-30.5) |
| Carer | 25.9 (22.9-29.2) | 34.6 (29.0-40.7) | 24.0 (19.3-29.3) | 22.6 (14.4-33.7) | 13.4 (8.0-21.5) | | 26.0 (23.8-28.4) |
|  | | | | | | | |

*Population estimates are presented as percentages or means, with 95% confidence intervals. Comparisons may be considered statistically significant when confidence intervals do not overlap .*

| TABLE 13. VISITS TO GENERAL PRACTITIONER | | | | | | |
| --- | --- | --- | --- | --- | --- | --- |
|  | Schizophrenia | Schizoaffective disorder | Bipolar disorder  with psychosis | Depressive psychosis | Delusional and other nonorganic psychoses | Any psychosis |
|  | N=857 | N=293 | N=319 | N=81 | N=92 | N=1642 |
|  | % (95%CI) | % (95% CI) | % (95% CI) | % (95% CI) | % (95% CI) | % (95% CI) |
| **Visits to general practitioner, past year** |  |  |  |  |  |  |
| General practitioner visits: any | 87.9 (85.5-89.9) | 88.5 (84.0-91.9) | 91.1 (87.5-93.8) | 93.9 (84.5-97.8) | 88.6 (80.7-93.6) | 89.0 (87.3-90.4) |
| General practitioner visits: mental health | 47.3 (43.8-50.9) | 52.1 (46.0-58.1) | 57.9 (52.2-63.4) | 70.0 (58.6-79.4) | 49.8 (39.0-60.6) | 51.5 (48.9-54.0) |
| General practitioner visits: physical health | 73.5 (70.3-76.5) | 78.4 (73.0-82.9) | 79.5 (74.5-83.8) | 84.2 (73.4-91.2) | 72.8 (62.3-81.3) | 76.0 (73.8-78.1) |
| No. of visits to GP if any, past year (mean) | 8 (8-9) | 10 (9-12) | 9 (8-10) | 12 (9-14) | 6 (5-7) | 9 (8-9) |
|  |  |  |  |  | | |

*Population estimates are presented as percentages or means, with 95% confidence intervals. Comparisons may be considered statistically significant when confidence intervals do not overlap .*

| TABLE 14. PHYSICAL EXAMINATIONS | | | | | | |
| --- | --- | --- | --- | --- | --- | --- |
|  | Schizophrenia | Schizoaffective disorder | Bipolar disorder  with psychosis | Depressive psychosis | Delusional and other nonorganic psychoses | Any psychosis |
|  | N=857 | N=293 | N=319 | N=81 | N=92 | N=1642 |
|  | % (95%CI) | % (95% CI) | % (95% CI) | % (95% CI) | % (95% CI) | % (95% CI) |
| **Physical examinations** |  |  |  |  |  |  |
| *In past year:* |  |  |  |  |  |  |
| Blood pressure measurement | 85.2 (82.4-87.5) | 86.6 (82.0-90.1) | 87.9 (83.7-91.2) | 86.5 (76.6-92.6) | 83.8 (74.5-90.2) | 85.9 (84.1-87.6) |
| Waist or weight measurement | 78.3 (75.2-81.1) | 77.6 (72.2-82.3) | 76.5 (71.3-81.1) | 75.1 (64.1-83.6) | 72.8 (62.0-81.4) | 77.4 (75.1-79.4) |
| Physical examination | 68.5 (65.1-71.6) | 67.6 (61.7-73.1) | 68.2 (62.6-73.4) | 74.5 (63.6-83.0) | 64.4 (53.4-74.0) | 68.3 (65.9-70.7) |
| Blood tests | 65.5 (62.1-68.8) | 62.7 (56.6-68.4) | 61.7 (55.9-67.1) | 70.5 (59.1-79.8) | 62.0 (51.1-71.8) | 64.4 (61.9-66.8) |
| Dental examination | 45.6 (42.1-49.2) | 47.0 (41.0-53.1) | 52.9 (47.1-58.6) | 49.3 (38.0-60.7) | 39.1 (29.1-50.1) | 47.1 (44.5-49.7) |
| X-ray or scan | 38.5 (35.1-42.0) | 46.0 (40.0-52.0) | 44.2 (38.5-50.0) | 60.0 (48.4-70.7) | 31.2 (22.1-42.0) | 41.5 (39.0-44.1) |
| Eye test | 34.0 (30.7-37.5) | 35.7 (30.1-41.7) | 38.9 (33.4-44.6) | 34.9 (24.8-46.6) | 30.9 (21.8-41.8) | 35.1 (32.7-37.6) |
| Hearing test | 10.4 (8.5-12.8) | 11.9 (8.6-16.4) | 9.2 (6.4-13.0) | 11.7 (5.8-22.2) | 11.8 (6.3-21.2) | 10.6 (9.1-12.3) |
| *In past two years:* |  |  |  |  |  |  |
| Bowel examination | 12.6 (10.4-15.2) | 11.9 (8.4-16.6) | 18.1 (14.0-23.1) | 28.6 (19.4-40.) | 16.3 (9.5-26.7) | 14.6 (12.8-16.6) |
| Cervical smear (women only) | 52.6 (46.0-59.2) | 57.6 (48.7-66.0) | 55.8 (47.9-63.3) | 57.5 (42.6-71.2) | 63.1 (42.8-79.5) | 55.3 (51.2-59.4) |
| Mammogram (women only) | 22.9 (17.8-29.1) | 25.1 (18.2-33.5) | 22.5 (16.7-29.6) | 21.5 (11.9-35.7) | 34.6 (18.5-55.2) | 23.6 (20.3-27.3) |
| Prostate cancer screen (men only) | 13.1 (10.4-16.3) | 16.2 (10.7-23.9) | 22.7 (15.9 -31.3) | 23.3 (10.7-43.4) | 14.9 (7.9-26.4) | 15.4 (13.1-18.0) |
|  |  |  |  |  | | |

*Population estimates are presented as percentages or means, with 95% confidence intervals. Comparisons may be considered statistically significant when confidence intervals do not overlap .*

# References

**Alberti, K. G., Eckel, R. H., Grundy, S. M., Zimmet, P. Z., Cleeman, J. I., Donato, K. A., Fruchart, J. C., James, W. P., Loria, C. M. & Smith, S. C.** (2009). Harmonizing the metabolic syndrome: a joint interim statement of the International Diabetes Federation Task Force on Epidemiology and Prevention; National Heart, Lung, and Blood Institute; American Heart Association; World Heart Federation; International Atherosclerosis Society; and International Association for the Study of Obesity. *Circulation* **120**, 1640-1645.

**Alonzo, T. A., Pepe, M. S. & Lumley, T.** (2003). Estimating disease prevalence in two-phase studies. *Biostatistics* **4**, 313-326.

**Australian Bureau of Statistics** (2008a). Australian Census Analytic Program: Counting the Homeless 2006. Catalogue No. 2050.0. Australian Bureau of Statistics: Canberra.

**Australian Bureau of Statistics** (2008b). National Survey of Mental Health and Wellbeing 2007. Catalogue No. 4326.0. Australian Bureau of Statistics: Canberra.

**Babor, T., de la Fuente, J., Saunders, J. & Grant, M.** (1992). *AUDIT: The alcohol use disorders identification test.* World Health Organization: Geneva.

**Craig, C. L., Marshall, A. L., Sjöström, M., Bauman, A. E., Booth, M. L., Ainsworth, B. E., Pratt, M., Ekelund, U., Yngve, A., Sallis, J. F. & Oja, P.** (2003). International physical activity questionnaire: 12-country reliability and validity. *Med Sci Sports Exerc.* **35**, 1381–95. doi: 10.1249/01.MSS.0000078924.61453.FB.

**Heatherton, T. F., Kozlowski, L. T., Frecker, R. C. & Fagerström, K.-O.** (1991). The Fagerström Test for Nicotine Dependence: a revision of the Fagerström Tolerance Questionnaire. *British Journal of Addiction to Alcohol and Other Drugs* **86**, 1119-1127.

**Jablensky, A. J., Kirkbride, J. B. & Jones, P. B.** (2011). The epidemiological horizon. In *Schizophrenia* (ed. D. R. Weinberger and P. J. Harrison), pp. 185-225. Wiley-Blackwell: Oxford.

**Jaeger, J., Berns, S. M. & Czobor, P.** (2003). The multidimensional scale of independent functioning: a new instrument for measuring functional disability in psychiatric populations. *Schizophrenia Bulletin* **29**, 153-68.

**Julious, S. A.** (2004). Using confidence intervals around individual means to assess statistical significance between two means. *Pharmaceutical Statistics*  **3**, 217-222.

**Kessler, R. C., Angermeyer, M., Anthony, J. C., De Graaf, R., Demyttenaere, K., Gasquet, I., De Girolamo, G., Semyon Gluzman, Gureje, O., Haro, J. M., Kawakami, N., Karam, A., Levinson, D., Mora, M. E. M., Oakley Browne, M. A., Posada-Villa, J., Stein, D. J., Tsang, C. H. A., Aguilar-Gaxiola, S., Alonso, J., Lee, S., Heeringa, S., Pennell, B.-E., Berglund, P., Gruber, M. J., Petukhova, M., Chatterji, S., Üstün, T. B. & on behalf of the WHO World Mental Health Survey Consortium** (2007). Lifetime prevalence and age-of-onset distributions of mental disorders in the World Health Organization's World Mental Health Survey Initiative. *World Psychiatry* **6**, 168–176.

**Morgan, V. A., Waterreus, A., Jablensky, A., Mackinnon, A., McGrath, J. J., Carr, V., Bush, R., Castle, D., Cohen, M., Harvey, C., Galletly, C., Stain, H. J., Neil, A. L., McGorry, P., Hocking, B., Shah, S. & Saw, S.** (2012). People living with psychotic illness in 2010: The second Australian national survey of psychosis. *Australian and New Zealand Journal of Psychiatry* **46**, 735-752.

**National Institutes of Health** (2000). The Practical Guide to the Identification, Evaluation and Treatment of Overweight and Obesity in Adults. National Institutes of Health: Bethesda.

**Nelson, H. E. & Willison, J.** (1991). *National Adult Reading Test (NART): Test Manual. Second Edition.* NFER Nelson: Windsor, UK.

**Randolph, C.** (1998). *Repeatable Battery for the Assessment of Neuropsychological Status (RBANS): Test Manual*. Harcourt Brace and Company: San Antonio.

**World Health Organization** ( 2000). Obesity: preventing and managing the global epidemic. Report of a WHO Consultation. WHO Technical Report Series 894. World Health Organization: Geneva.
